# The endonuclease domain of the LINE-1 ORF2 protein can tolerate multiple mutations

**DOI:** 10.1186/s13100-016-0064-x

**Published:** 2016-04-19

**Authors:** Kristine J. Kines, Mark Sokolowski, Dawn L. deHaro, Claiborne M. Christian, Melody Baddoo, Madison E. Smither, Victoria P. Belancio

**Affiliations:** Department of Structural and Cellular Biology, Tulane School of Medicine, Tulane Cancer Center and Tulane Center for Aging, New Orleans, LA 70112 USA

**Keywords:** LINE-1, L1, ORF2, Endonuclease, Mutation, Retrotransposition, Phosphorylation

## Abstract

**Background:**

Approximately 17 % of the human genome is comprised of the *L*ong *IN*terspersed *E*lement-1 (LINE-1 or L1) retrotransposon, the only currently active autonomous family of retroelements. Though L1 elements have helped to shape mammalian genome evolution over millions of years, L1 activity can also be mutagenic and result in human disease. L1 expression has the potential to contribute to genomic instability via retrotransposition and DNA double-strand breaks (DSBs). Additionally, L1 is responsible for structural genomic variations induced by other transposable elements such as Alu and SVA, which rely on the L1 ORF2 protein for their propagation. Most of the genomic damage associated with L1 activity originates with the endonuclease domain of the ORF2 protein, which nicks the DNA in preparation for target-primed reverse transcription.

**Results:**

Bioinformatic analysis of full-length L1 loci residing in the human genome identified numerous mutations in the amino acid sequence of the ORF2 endonuclease domain. Some of these mutations were found in residues which were predicted to be phosphorylation sites for cellular kinases. We mutated several of these putative phosphorylation sites in the ORF2 endonuclease domain and investigated the effect of these mutations on the function of the full-length ORF2 protein and the endonuclease domain (ENp) alone. Most of the single and multiple point mutations that were tested did not significantly impact expression of the full-length ORF2p, or alter its ability to drive Alu retrotransposition. Similarly, most of those same mutations did not significantly alter expression of ENp, or impair its ability to induce DNA damage and cause toxicity.

**Conclusions:**

Overall, our data demonstrate that the full-length ORF2p or the ENp alone can tolerate several specific single and multiple point mutations in the endonuclease domain without significant impairment of their ability to support Alu mobilization or induce DNA damage, respectively.

**Electronic supplementary material:**

The online version of this article (doi:10.1186/s13100-016-0064-x) contains supplementary material, which is available to authorized users.

## Background

The *L*ong *IN*terspersed *E*lement-1 (LINE-1 or L1) retrotransposon is the only currently active autonomous non-LTR retroelement in the human genome. Most of the approximately 500,000 L1 loci have been truncated or mutated and are incapable of further retrotransposition [1, 2]. L1 is required for the mobilization of non-autonomous retrotransposons such as Alu and SVA elements [3, 4], and together these retroelements comprise about a third of the human genome [2]. The fully functional L1 element encodes two proteins (ORF1p and ORF2p), both of which are required for its retrotransposition [5]. ORF1p is an RNA-binding protein with nucleic chaperone activity [6, 7] and ORF2p is a multifunctional protein with endonuclease and reverse transcriptase enzymatic activities and a cysteine-rich domain [8, 9]. L1 and L1-driven retroelements amplify through a copy-and-paste mechanism, resulting in *de novo* insertions in the genome. After integration, these retroelements can interfere with gene expression [10, 11] or serve as substrates for non-allelic homologous recombination events, which can lead to disease-relevant genomic deletions, inversions or translocations [12–22].

L1 and its Alu and SVA parasites have significantly impacted the human genome for better or worse (reviewed [23]). L1 has had a major role in generating structural variations in the host genome through retrotransposition and post-insertional genomic rearrangements [24]. However, expression of functional L1 loci can potentially be detrimental at the cellular or organismal level. Transient expression of L1 in mammalian cells results in L1 retrotransposition, and the generation of DNA double-strand breaks (DSBs) [25, 26]. This genomic damage can be significant and lead to apoptosis or senescence [27, 28]. Both L1 retrotransposition and L1-induced DSBs result from the activity of the endonuclease domain present in the ORF2 protein, which functions to introduce nicks into the cellular DNA in preparation for L1 integration via target-primed reverse transcription [8, 29]. Mutations within the endonuclease domain have the potential to impair endonuclease activity and thereby diminish subsequent L1-induced genomic damage. Aside from a few critical residues that have been characterized [5, 8, 30, 31], it is not known how well the endonuclease domain can tolerate mutations in terms of maintaining its function.

In this study, we analyzed full-length L1 loci within the human genome to identify naturally occurring mutations within the ORF2 endonuclease domain. Some of these mutated residues are evolutionarily conserved or presumed to be structurally important [8, 31]. Moreover, several of these mutated residues were predicted to be phosphorylation sites for various cellular kinases. Recent studies reported that the phosphorylation of ORF1p is required for L1 retrotransposition [32] and that these and other putative phosphorylation sites within ORF1p may be involved in the regulation of L1 activity by melatonin receptor signaling [33]. These studies raise the question of whether ORF2p could also be post-translationally modified. Focusing on putative phosphorylation sites, as a way to investigate a manageable subset of positions with possible biological relevance to L1 function, we mutated several amino acid residues within the ORF2 endonuclease domain and investigated the effect of these mutations on endonuclease activity. We characterized the ability of the mutated ENp and ORF2p to cause toxicity, induce H2AX phosphorylation (a marker for DNA DSBs [34, 35]), and drive Alu retrotransposition. Our findings demonstrate that all of the tested individual mutations, as well as most of the various combinations of these mutations, are tolerated without significantly impacting the L1 ORF2 endonuclease function, either in the context of the full-length ORF2p or in the ENp alone.

## Results

### Identification of mutations in the endonuclease domain of full-length human L1 loci

**Fig. 1:**
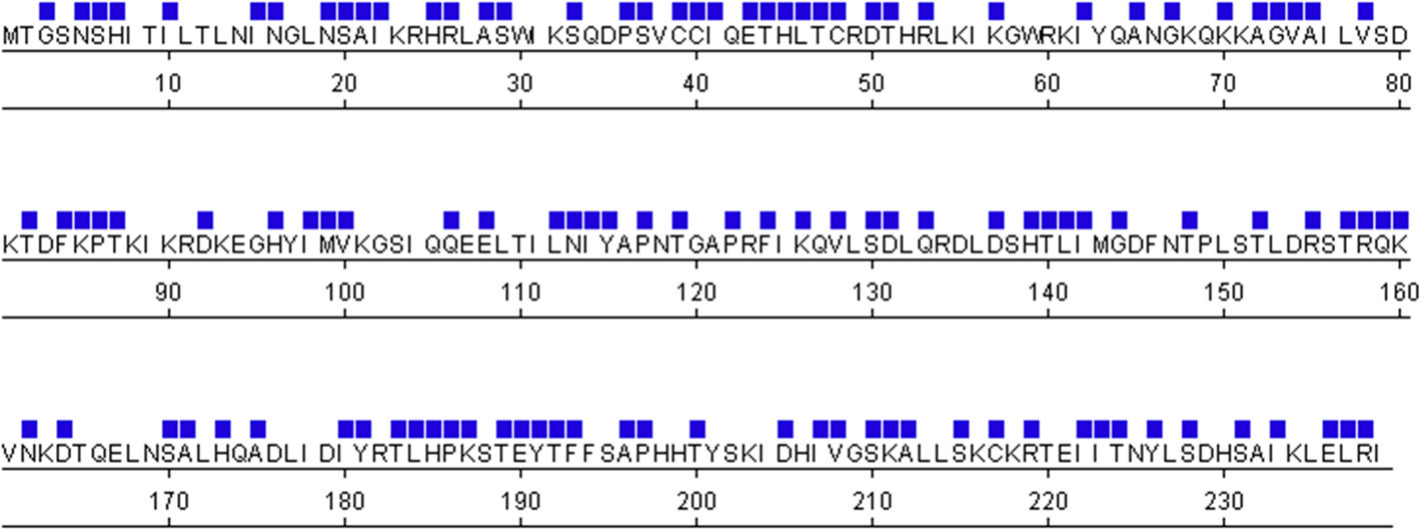
Mutations in the ORF2 endonuclease domain from full-length L1 loci in the human genome. Bioinformatic analysis using L1Base [36] revealed numerous mutations in the ORF2 endonuclease domains of 134 intact, full-length L1 loci. Positions of mutations relative to the sequence of the L1.3 ORF2 endonuclease domain are indicated by a blue square above the amino acid residue

Using L1Base [36], we analyzed the sequences of full-length L1 loci present in the human genome to identify naturally occurring mutations in the ORF2 endonuclease domain. Our search parameters were set to identify L1 loci that contain intact ORF1 and ORF2 sequences (no gaps, premature stops or frameshifts). We identified 134 L1 loci that satisfy these search criteria, the majority of which were L1Ta and L1PA2 families (Additional file 1: Table S1). None of the 134 full-length L1 loci fitting our search criteria had any mutations at amino acid H230 and only one locus contained a mutation at D205 (Fig. 1; Additional file 1: Table S1). These two residues are absolutely critical for ORF2 endonuclease function [8, 31]. The maximum number of mutations found in the endonuclease domain of any of the 134 full-length L1 loci was 11. Aligning the ORF2 protein sequences of the full-length L1 loci extracted from L1Base against the active human L1.3 element revealed that 118 of the 239 amino acids in the endonuclease domain were mutated at least once (Fig. 1; Additional file 2: Figure S1). However, three of these mutations may be specific to the L1PA2 sequence (I15V, A21P, V208L).

The large number of naturally occurring mutations prompted us to narrow our focus on a specific subset of mutated positions within the ORF2 endonuclease. Our bioinformatic analysis identified multiple mutations of serines and threonines in the endonuclease domain, which may be of particular interest as these amino acids are commonly phosphorylated by cellular kinases [37]. We utilized the ELM prediction tool [38] to identify several short linear motifs within the endonuclease domain that were expected to be recognized by serine/threonine kinases. We also used the NetPhos 2.0 prediction program to identify amino acids having a high probability of being phosphorylated [39]. The following amino acids were predicted to be phosphorylated by both programs: S29, S33, S37, S79, T82, S151, S188, T189, T220, T224 and S228 (Additional file 3: Table S2). Additionally, ELM and NetPhos 2.0 identified two very high probability residues just outside of the endonuclease domain (S312 and S335). To evaluate the evolutionary conservation of the putative phosphorylation sites identified by the prediction programs, we aligned the amino acid sequence of the L1 ORF2 endonuclease domain from eight representative species within the Supraprimate clade of mammals (Additional file 4: Figure S2). With respect to the human L1 sequence, S37, S188 and T189 were moderately conserved (present in 50 % or more of the investigated species), while S79, S151 and S228 were highly conserved (present in almost all investigated species). Even the least conserved residues were still shared among the hominid clade of primates (humans, chimpanzees and bonobos).

### Mutations of several putative phosphorylation sites in the ORF2 endonuclease domain did not alter its ability to drive Alu retrotransposition, cause toxicity, or impact expression of the full-length ORF2p

**Fig. 2:**
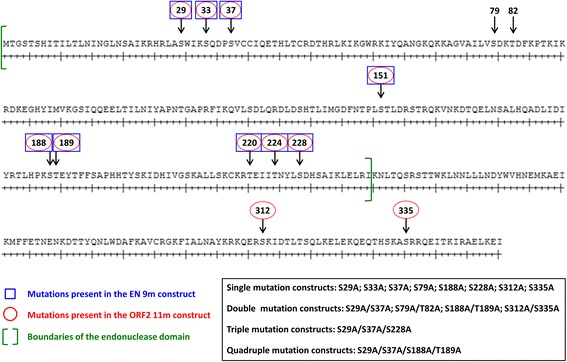
Schematic representation of the putative phosphorylation sites within ORF2 mutated in this study. Numbered arrows indicate the locations of the putative phosphorylation sites mutated in this study. The boundaries of the ORF2 endonuclease domain are indicated with green brackets. Plasmids encoding for the full-length ORF2p were generated with the following mutations: S29A; S33A; S37A; S79A; S188A; S228A; S29A/S37A; S79A/T82A; S188A/T189A; S29A/S37A/S228A; S29A/S37A/S188A/T189A; S312A; S335A; and S312A/S335A. ORF2 11m contains the following mutations (*red ovals*): S29A/S33A/S37A/S151A/S188A/T189A/T220A/T224A/S228A/S312A/S335A. Plasmids encoding for the endonuclease domain (ENp) alone were generated with the following mutations: S29A; S33A; S37A; S79A; S188A; S228A; S29A/S37A; S79A/T82A; S188A/T189A; S29A/S37A/S228A; and S29A/S37A/S188A/T189A. EN 9m contains the following mutations (*blue boxes*): S29A/S33A/S37A/S151A/S188A/T189A/T220A/T224A/S228A. Plasmids encoding for the full-length L1 containing the following mutations within ORF2 were generated: S29A; S33A; S312A; S335A; and S312A/S335A

As our main interest is in understanding the impact of mutations on L1 endonuclease function, we chose to investigate the functional impact of mutations in putative phosphorylation sites because, if found relevant, these sites could also play a regulatory role. Amino acid residues that were scored by both the ELM and NetPhos prediction programs were selected for mutagenesis (Additional file 3: Table S2). We generated expression plasmids containing codon-optimized human L1 ORF2 sequence with either serine to alanine (S to A) or threonine to alanine (T to A) point mutations in putative phosphorylation sites (Fig. 2). The resulting plasmids contained one (S29A; S33A; S37A; S79A; S188A; S228A), two (S29A/S37A; S79A/T82A; S188A/T189A), three (S29A/S37A/S228A), or four (S29A/S37A/S188A/T189A) point mutations in putative phosphorylation sites within the endonuclease domain. The ORF2 11m construct was designed to include mutations in sites predicted to be phosphorylated by kinases in the CMGC group (*C*DK, *M*APK, *G*SK3 and *C*LK) [40]. ORF2 11m contains 11 mutations (S29A/S33A/S37A/S151A/S188A/T189A/T220A/T224A/S228A/S312A/S335A); 9 mutations are in the putative phosphorylation sites within the endonuclease domain and the remaining 2 are located between the endonuclease and z-motif region. Because these mutant constructs were tested in transiently transfected HeLa and 293 cells, we used an NGS RNAseq approach to confirm that these cell lines express many cellular kinases (Additional file 5: Table S3) [41].

**Fig. 3:**
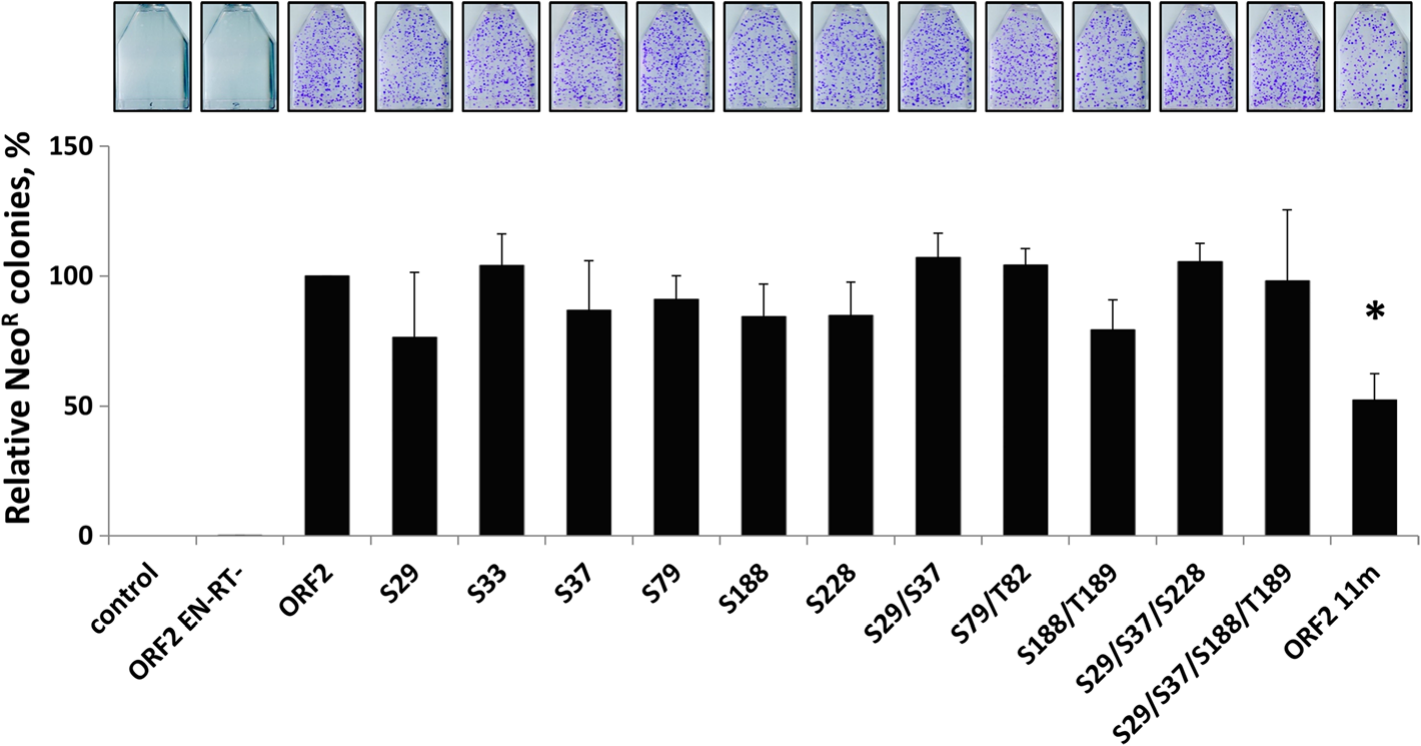
Alu retrotransposition driven by ORF2 proteins containing mutations in putative phosphorylation sites. ORF2 proteins containing mutations in the indicated putative phosphorylation sites were used to drive Alu retrotransposition in HeLa cells, as previously described [3]. ORF2 is the functional protein and ORF2 EN-RT- is a non-functional protein containing mutations in the endonuclease (D205A) and reverse transcriptase (D702A) domains. Control indicates cells transfected with an empty vector and the Alu retrotransposition reporter plasmid. The graph depicts the relative number of Alu retrotransposition events as represented by Neo^R^ colonies (Y-axis). Asterisks indicate a statistically significant difference in Alu retrotransposition compared to ORF2 (*t*-test, *P* ≤ 0.05)

As an indication of protein function, we investigated the ability of the ORF2 proteins containing the above described mutations to mobilize Alu using a previously described retrotransposition assay [3]. With the exception of the ORF2 11m mutant, all of the full-length mutant ORF2 proteins supported Alu retrotransposition at similar levels to the functional ORF2p (*t*-test, *P* ≥ 0.05) when transiently expressed in HeLa cells (Fig. 3). A significant ~50 % decrease in retrotransposition was observed when Alu mobilization was driven by the ORF2 11m mutant protein (*t*-test, *P* ≤ 0.05).

**Fig. 4:**
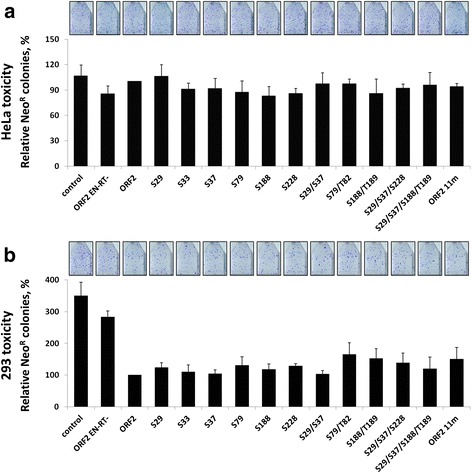
Acute toxicity assay in HeLa and 293 cells transiently transfected with ORF2 putative phosphorylation mutant plasmids. **a** HeLa cells were cotransfected with a Neo^R^ expression vector and the indicated ORF2 putative phosphorylation mutant plasmid. **b** 293 cells were cotransfected with a Neo^R^ expression vector and the indicated ORF2 putative phosphorylation mutant plasmid. In both panel **a** and **b** ORF2 is the functional protein and ORF2 EN-RT- is a non-functional protein containing mutations in the endonuclease (D205A) and reverse transcriptase (D702A) domains. Control indicates cells transfected with an empty vector and the Neo^R^ expression vector. Colony formation was assayed after 2 weeks under G418 selection (Y-axis) and used as a measure of toxicity as previously described [26, 42]

In addition to genomic damage due to retrotransposition, expression of the ORF2 protein can cause cellular toxicity in a dose-dependent manner when ectopically expressed at high levels [26, 27]. A positive or negative change in ORF2p toxicity may mask or cause subsequent variations in ORF2p-driven Alu mobilization. Using a previously described assay [26], we measured acute toxicity following transient transfection of the ORF2 putative phosphorylation plasmids to determine if variations in cellular toxicity may contribute to the observed reduction in Alu retrotransposition driven by the ORF2 11m protein (Additional file 6: Figure S3A). For this reason, the same amount of DNA that was transfected in the retrotransposition assay was used for evaluation of potential changes in ORF2p toxicity. Results in Fig. 4a demonstrate that there were no significant differences in toxicity between the functional ORF2p and any of the putative phosphorylation mutant proteins after transient expression in HeLa cells. In contrast to previously reported results [26], we did not observe any toxicity associated with the expression of functional or mutant ORF2 proteins in HeLa cells under our experimental conditions. This discrepancy is likely due to the 20-fold difference in the amount of plasmid DNA transfected per cell between the reported transfection conditions (2 μg plasmid per 100,000 cells in a 6-well plate) and the conditions used here (0.5 μg per 500,000 cells in a T75 flask). Additionally, it was reported that expression of the full-length ORF2p alone was not as efficient in generating γH2AX foci in HeLa cells as was the expression of the full-length L1 [26]. We did observe toxicity after transient expression of the functional ORF2p and putative phosphorylation mutant proteins in 293 cells (Fig. 4b). Consistent with the results obtained in HeLa cells, no significant differences between the toxicity observed after expression of the functional and mutated ORF2 proteins were detected.

**Fig. 5:**
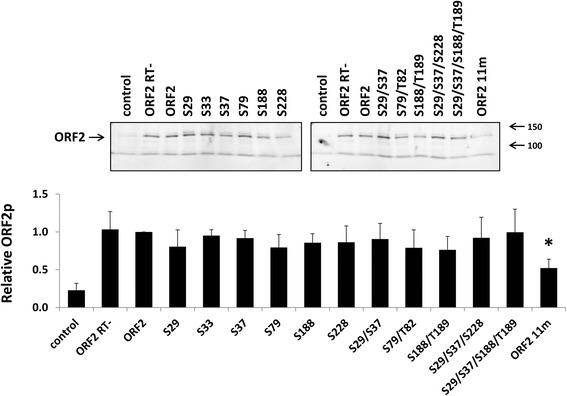
Expression and detection of ORF2 proteins containing mutations in putative phosphorylation sites. Top panel: Representative western blot analysis of total cell lysates harvested from HeLa cells transfected with the indicated ORF2 putative phosphorylation mutant constructs. ORF2 is the functional protein and ORF2 RT- is a non-functional protein containing a mutation in the reverse transcriptase (D702A) domain. Control lanes indicate cells transfected with an empty vector. Lysates were probed with polyclonal antibodies generated against the human L1 ORF2 protein. Bottom panel: Western blot quantitation. For each sample, the signal detected for ORF2p was normalized to the total protein load. These relative numbers were expressed as a proportion of the relative number detected from the functional ORF2p. Asterisk denotes a significant difference in the steady-state levels relative to the functional ORF2p (*t*-test, *P* ≤ 0.05)

To determine whether the significant reduction in Alu retrotransposition driven by the ORF2 11m mutant was a result of altered protein expression, we analyzed total protein lysates harvested from HeLa cells transiently transfected with each of the ORF2 mutant plasmids described above. Western blot analysis using antibodies specific to the human L1 ORF2 protein [42, 43] detected ORF2p in the total lysates of transfected HeLa cells (Fig. 5). Quantitation of the relative ORF2p expression levels revealed an approximately 50 % reduction in the steady-state levels of the ORF2 11m protein in comparison to the functional ORF2p. No statistically significant differences in expression were observed between the functional ORF2p and any of the other ORF2 proteins containing mutations in putative phosphorylation sites. The same lysates were also probed with anti-γH2AX antibodies, as histone H2AX is phosphorylated in response to DNA DSBs and can therefore be used as an indication of DNA damage (Additional file 7: Figure S4) [34]. Consistent with our toxicity results in HeLa cells, expression of the functional ORF2p or any of the ORF2 putative phosphorylation mutant proteins generated γH2AX signals that were not significantly different than the background signal observed with the empty vector control or non-functional ORF2 protein (*t*-test, *P* ≥ 0.05).

### Mutations of several putative phosphorylation sites in the ORF2 endonuclease domain did not alter ENp expression or its ability to induce DNA damage and cause toxicity

Previous in vitro studies have suggested that endonuclease function is repressed in the full-length ORF2 protein [29]. We recently reported that the endonuclease domain of human L1 ORF2p is stable and functional when expressed in mammalian cells [43], which enabled us to characterize the effects of the putative phosphorylation site mutations on the function of the endonuclease domain (ENp) independent of the full-length ORF2p. We generated plasmids containing the sequence of the endonuclease domain (amino acids 1–239) with one (S29A; S33A; S37A; S79A; S188A; S228A), two (S29A/S37A; S79A/T82A; S188A/T189A), three (S29A/S37A/S228A), or four (S29A/S37A/S188A/T189A) point mutations in putative phosphorylation sites (Fig. 2). The EN 9m plasmid includes the following mutations, all of which are also contained in the ORF2 11m construct: S29A/S33A/S37A/S151A/S188A/T189A/T220A/T224A/S228A.

**Fig. 6:**
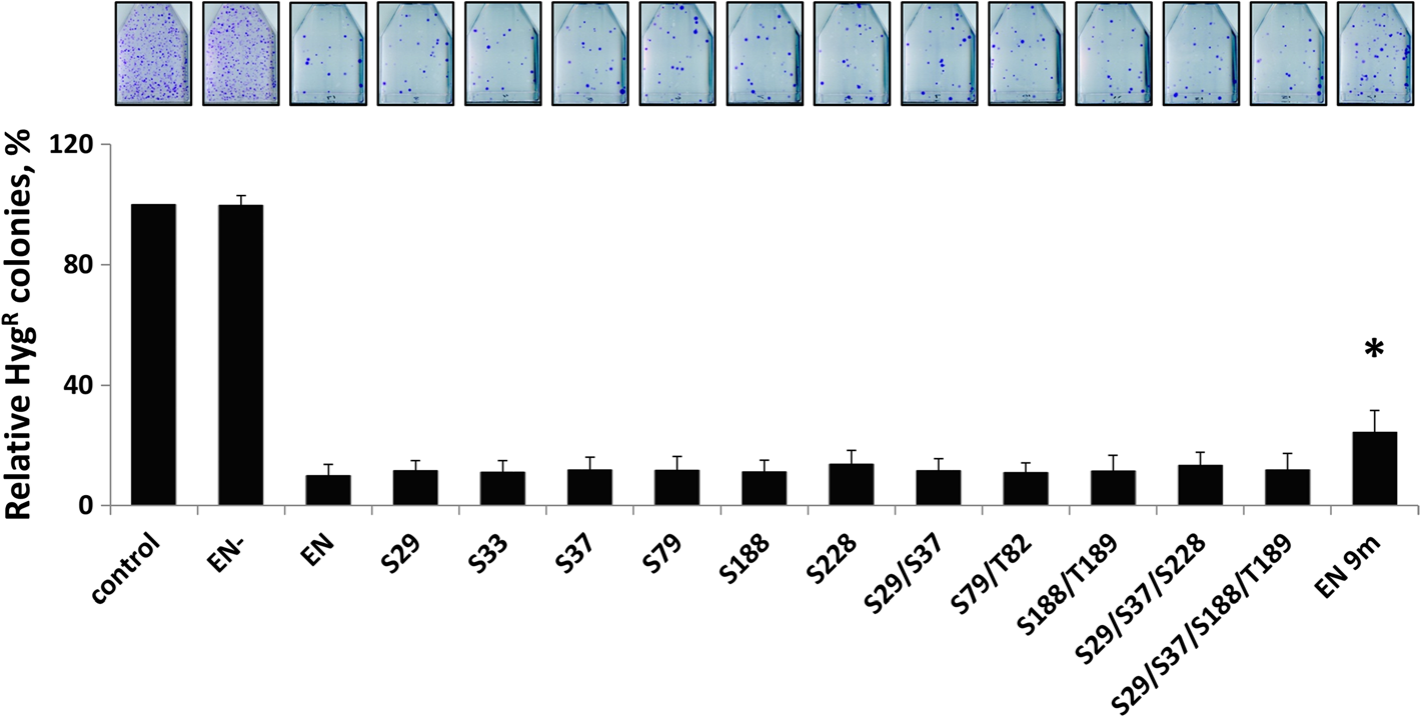
Chronic expression of EN proteins containing mutations in putative phosphorylation sites causes toxicity. HeLa cells were transfected with a single expression plasmid containing both Hygro^R^ and the indicated EN putative phosphorylation mutant sequence. Hygromycin selection was maintained for 2 weeks post-transfection, allowing stable expression of ENp throughout the assay. EN is the functional protein and EN- is a non-functional protein containing inactivating mutations (D205A/H230A). Control indicates cells transfected with an empty vector. Colony formation was assayed after 2 weeks under hygromycin (Y-axis) and used as a measure of toxicity as previously described [27, 43]. Asterisks indicate a statistically significant difference in the relative number of Hygro^R^ colonies compared to EN (*t*-test, *P* ≤ 0.05)

Using a previously reported assay [27, 43], we measured chronic toxicity of these constructs in HeLa cells (Additional file 6: Figure S3B; Fig. 6). With the exception of the EN 9m mutant, all of the mutant endonuclease proteins were as toxic as the functional ENp. Chronic expression of the EN 9m mutant protein resulted in a statistically significant 2.5-fold difference in the relative colony number in comparison to the functional ENp (Fig. 6). Similar results were obtained after transient transfection of HeLa cells with the EN mutant plasmids in an acute toxicity assay (Additional file 6: Figure S3A; Additional file 8: Figure S5).

**Fig. 7:**
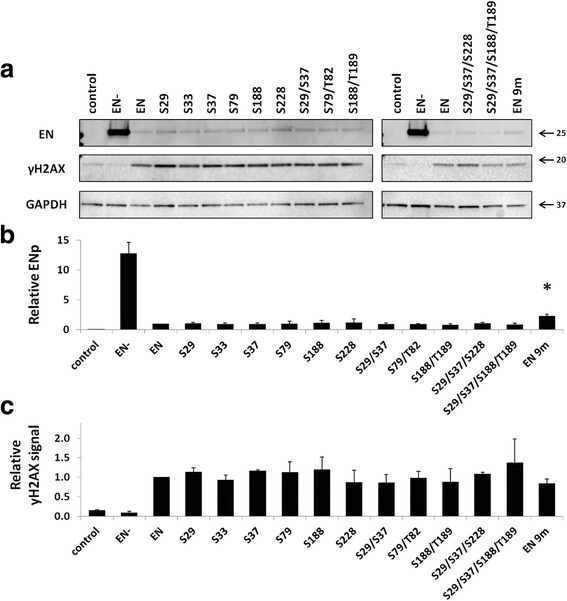
Expression of EN proteins containing mutations in putative phosphorylation sites can induce DNA damage. **a** Representative western blot analysis of total cell lysates harvested from HeLa cells transiently transfected with the indicated EN putative phosphorylation mutant plasmids. EN is the functional protein and EN- is a non-functional protein containing inactivating mutations (D205A/H230A). Control lanes indicate cells transfected with an empty vector. **a** Lysates were probed with polyclonal antibodies generated against the human L1 ORF2 endonuclease domain [42, 43], top panel; anti-γH2AX antibodies to detect the phosphorylation of histone H2AX in response to DNA damage, middle panel; and anti-GAPDH to serve as a loading control, bottom panel. **b** Western blot quantitation. For each sample, the signal detected for ENp was normalized to the signal detected for GAPDH. These relative numbers were expressed as a proportion of the relative number detected from the functional ENp. Asterisk denotes a significant difference in the steady-state levels relative to the functional ENp (*t*-test, *P* ≤ 0.05). **c** Western blot quantitation. For each sample, the signal detected for γH2AX was normalized to the signal detected for GAPDH. These relative numbers were expressed as a proportion of the relative number detected from the functional ENp

We have previously reported that expression of the endonuclease domain alone in cultured cells results in a DNA damage response [43]. With the exception of the EN 9m mutant, which was roughly 2-fold higher than the functional ENp, steady-state expression levels were comparable between the functional and mutant proteins (Fig. 7). As previously reported [43], we detected higher steady-state levels of the non-functional ENp (EN-) in comparison to the functional ENp. Similar results were observed with western blot analysis of total protein lysates harvested from 293 cells transiently transfected with the EN expression plasmids (Additional file 9: Figure S6). Western blot analysis detected a γH2AX signal in the total protein lysates from HeLa cells transiently transfected with each of the EN mutant constructs. This result demonstrates that these mutant EN proteins are capable of inducing a DNA damage response (Fig. 7c). Quantitation of the relative γH2AX signals showed that expression of the functional ENp and the EN putative phosphorylation mutant proteins triggered similar levels of H2AX phosphorylation in HeLa cells (i.e., no statistically significant difference was detected) (Fig. 7c).

### Mutations of selected putative phosphorylation sites outside of the endonuclease domain did not alter ORF2p expression or its ability to mobilize Alu, induce DNA damage and cause toxicity

In comparison to the functional ORF2p, expression of the ORF2 11m mutant protein resulted in a reduction in Alu retrotransposition, a decrease in steady-state expression levels and similar levels of toxicity (Figs. 3, 4, and 5). Expression of the EN 9m mutant protein resulted in an increase in steady-state expression levels and less toxicity compared to its functional ENp counterpart (Figs. 6 and 7). The observed differences in toxicity and expression may be due to the presence of the two additional mutations outside of the endonuclease domain in the ORF2 11m protein; alternatively, the nine mutations shared by the ORF2 11m and EN 9m mutants may affect protein function differently in the context of the endonuclease domain alone versus the full-length ORF2p. We generated ORF2 expression plasmids to investigate any independent functional effects of the S312A and S335A mutations located outside of the endonuclease domain (Fig. 2).

**Fig. 8:**
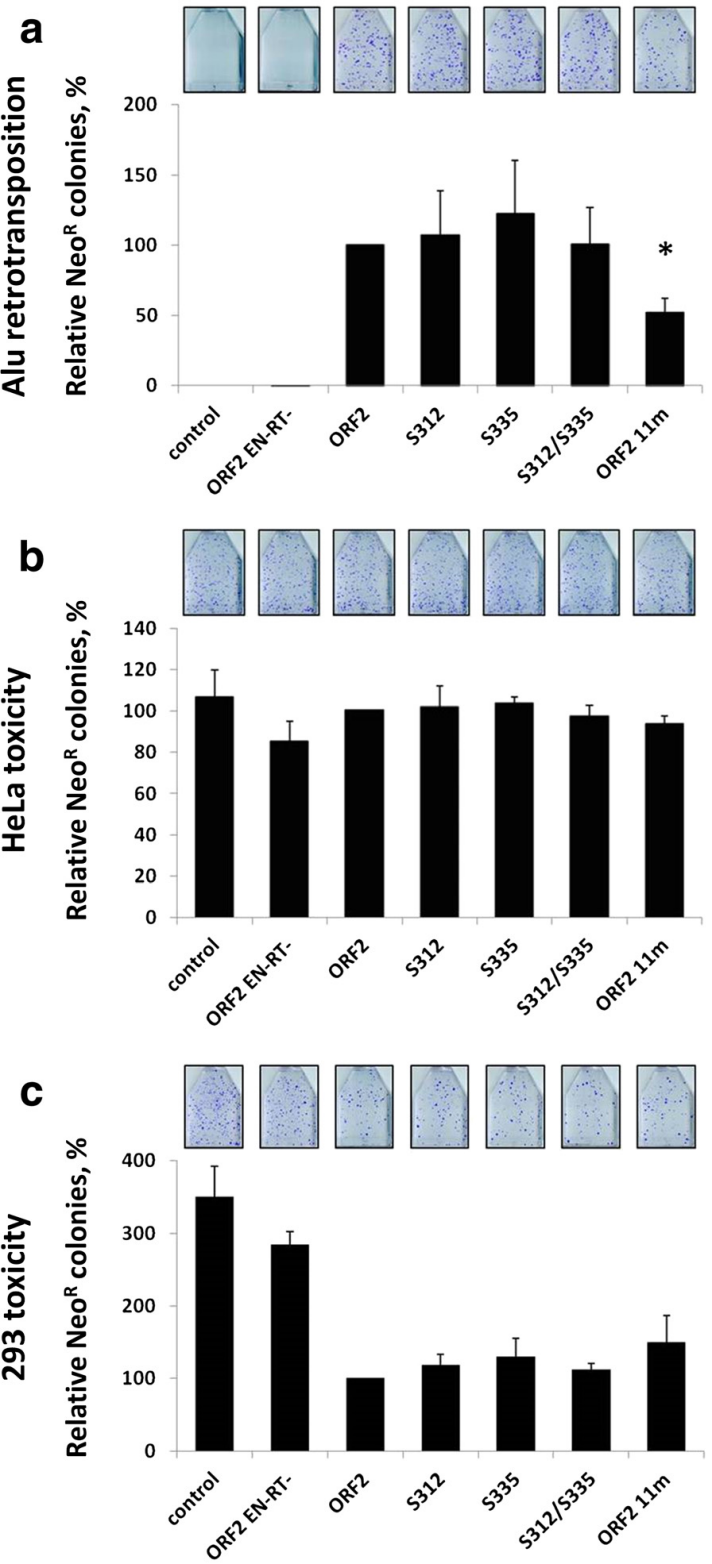
Analysis of select putative phosphorylation sites outside of the endonuclease domain. **a** Alu retrotransposition: ORF2 proteins containing mutations in the indicated putative phosphorylation sites were used to drive Alu retrotransposition in HeLa cells, as previously described [3]. ORF2 is the functional protein and ORF2 EN-RT- is a non-functional protein containing mutations in the endonuclease (D205A) and reverse transcriptase (D702A) domains. Control indicates cells transfected with an empty vector and the Alu retrotransposition reporter plasmid. The graph depicts the relative number of Alu retrotransposition events as represented by Neo^R^ colonies (Y-axis). Asterisks indicate a statistically significant difference in Alu retrotransposition compared to ORF2 (*t*-test, *P* ≤ 0.05). **b** Acute toxicity: HeLa cells were cotransfected with a Neo^R^ expression vector and the indicated ORF2 putative phosphorylation mutant plasmid. **c** Acute toxicity: 293 cells were cotransfected with a Neo^R^ expression vector and the indicated ORF2 putative phosphorylation mutant plasmid. In both panels **b** and **c**, ORF2 is the functional protein and ORF2 EN-RT- is a non-functional protein containing mutations in the endonuclease (D205A) and reverse transcriptase (D702A) domains. Control indicates cells transfected with an empty vector and the Neo^R^ expression vector. Colony formation was assayed after 2 weeks under G418 selection (Y-axis) and used as a measure of toxicity as previously described [26, 43]

**Fig. 9:**
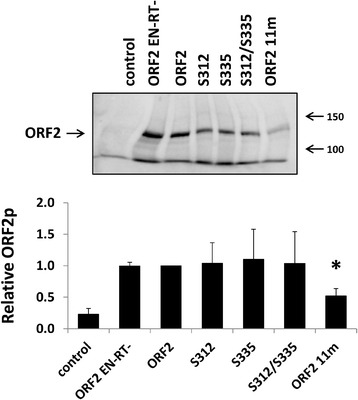
Expression of ORF2p containing mutations in selected putative phosphorylation sites outside of the endonuclease domain. Top panel: Representative western blot analysis of total cell lysates harvested from HeLa cells transfected with the indicated ORF2 putative phosphorylation mutant constructs. ORF2 is the functional protein and ORF2 EN-RT- is a non-functional protein containing mutations in the endonuclease (D205A) and reverse transcriptase (D702A) domains. Control lanes indicate cells transfected with an empty vector. Lysates were probed with polyclonal antibodies generated against the human L1 ORF2 protein. Bottom panel: Western blot quantitation. For each sample, the signal detected for ORF2p was normalized to the total protein load. These relative numbers were expressed as a proportion of the relative number detected from the functional ORF2p. Asterisk denotes a significant difference in the steady-state levels relative to the functional ORF2p (*t*-test, *P* ≤ 0.05)

In contrast to the ORF2 11m protein, ORF2 proteins containing only the S312A, S335A or S312A/S335A mutations supported Alu retrotransposition as efficiently as the functional ORF2p (Fig. 8a). Moreover, there were no significant differences in toxicity after transient expression of these proteins in HeLa and 293 cells (Fig. 8b and c). No statistically significant difference was found between the steady-state expression levels of the functional ORF2p and the ORF2 proteins containing either the individual mutations outside of the endonuclease domain or their combination (Fig. 9).

**Fig. 10:**
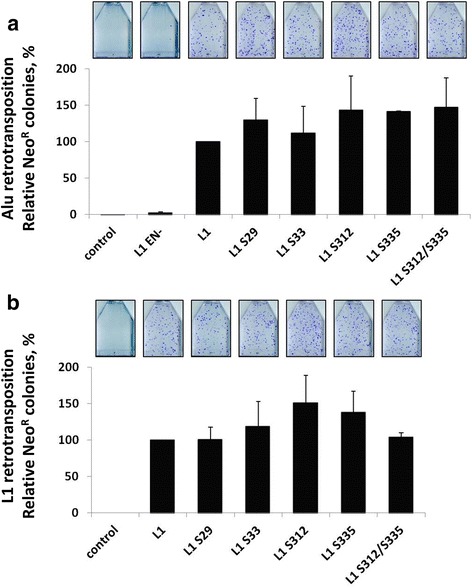
Retrotransposition driven by full-length L1 elements containing mutations in putative phosphorylation sites within ORF2. **a** Alu retrotransposition: Full-length L1 elements containing the indicated putative phosphorylation mutations within the ORF2 sequence were used to drive Alu retrotransposition in HeLa cells, as previously described [3]. L1 is the functional element and L1 EN- is a non-functional element containing a mutation in the ORF2 endonuclease domain (D205A). Control indicates cells transfected with an empty vector and the Alu retrotransposition reporter plasmid. The graph depicts the relative number of Alu retrotransposition events as represented by Neo^R^ colonies (Y-axis). **b** L1 retrotransposition: Full-length L1 elements containing the indicated putative phosphorylation mutations within the ORF2 sequence were used in an L1 retrotransposition assay in HeLa cells, as previously described [5, 56]. L1 is the functional element and control indicates cells transfected with an empty plasmid. The graph depicts the relative number of L1 retrotransposition events as represented by Neo^R^ colonies (Y-axis)

We also created L1 constructs containing select putative phosphorylation site mutations within the ORF2 sequence, in order to evaluate the effect of these mutations on ORF2p function in the context of the full-length L1. Consistent with the results obtained with the ORF2p expression plasmids, all of the full-length L1 mutants were as efficient as the functional L1 in driving Alu retrotransposition, or their own mobilization, in HeLa cells (Fig. 10).

## Discussion

Most L1-induced genomic damage originates with the ORF2 endonuclease, as it initiates endonuclease-dependent retrotransposition events and its activity is implicated in the generation of L1-associated DNA DSBs [8, 26, 29]. The vast majority of full-length L1 loci have become inactive through the accumulation of post-insertional mutations [44]. Given the importance of endonuclease activity to retrotransposition and its relevance to human health, we examined the sequences of full-length L1 loci to find naturally occurring mutations in the endonuclease domain with the potential to affect its function. Our bioinformatic analysis of 134 full-length human L1 loci revealed that all but one locus have retained the wild-type amino acid at position 205 (D205), a residue that is confirmed to be important for endonuclease function (Additional file 2: Figure S1) [8]. Overall, our bioinformatic analysis identified 118 amino acid positions within the sequence of the endonuclease domain that were mutated relative to the L1.3 sequence. These mutations appear to be randomly distributed throughout the endonuclease sequence (Fig. 1).

Further investigation revealed that 25 % of the 118 mutated positions were serines, threonines, or tyrosines. Combined, serines, threonines and tyrosines comprise about 20 % of the functional endonuclease sequence. More than 50 % of all serine, threonine, or tyrosine residues present in the endonuclease domain were found to be mutated in at least one locus (11 of 20 serines; 15 of 21 threonines; 4 of 7 tyrosines). Most of these mutations did not appear to be due to the presence of CpG dinucleotides within the codons encoding serines and threonines, as there are only two CpG-containing codons (S47, T157) in the human ORF2 endonuclease domain. The underrepresentation of CpG-containing codons is not surprising, given the AT-richness of the L1 coding sequence [45, 46].

Identification of a subset of naturally occurring mutations with potential biological relevance to L1 endonuclease function provided a rationale for investigating the functional effect of mutations at some of these positions (Fig. 2; Additional file 3: Table S2). With the exception of the ORF2 11m mutant, all of the ORF2 proteins with single or multiple point mutations of putative phosphorylation sites behaved similarly to the functional ORF2p in terms of expression and ability to support Alu mobilization. We did not observe any additive effect on the ability of ORF2p to drive Alu retrotransposition as the number of mutations was increased from one to four (Fig. 3). Interestingly, the ORF2 11m protein, which contained 11 point mutations, was still able to drive fairly efficient Alu retrotransposition in HeLa cells, albeit at a 50 % lower level than the functional ORF2p (Fig. 3). This reduction is consistent with the 50 % decrease in the steady-state levels of its expression relative to the functional ORF2p (Fig. 5). Out of the 11 putative phosphorylation sites that were mutated in the ORF2 11m construct, only 3 sites (S151A, T220A and T224A) were not tested individually or in combination with any of the other 8 mutations for their ability to affect L1 ORF2p expression and retrotransposition. This raises the possibility that the reduction in ORF2 11m protein expression and its ability to drive Alu mobilization may be entirely due to one or more of those three mutations. Alternatively, a combination of all 11 mutations may be responsible for the observed effect. Regardless, the important finding remains that many mutations within the ORF2p endonuclease domain, individually or in various combinations, were tolerated by the enzyme. Despite containing as many as 11 mutations, the Alu retrotransposition potential was only reduced by 50 %.

As with the results from the mutant ORF2 proteins in the retrotransposition assays, we did not observe any additive effect on the ability of ENp to cause toxicity as the number of mutations was increased from one to four (Fig. 6, Additional file 8: Figure S5). The reduction in toxicity observed with the EN 9m mutant in comparison to the functional ENp may suggest that a threshold was crossed at a higher number of mutations. Alternatively, the 3 putative phosphorylation site mutations that were not tested individually (S151A, T220A and T224A) may have been responsible for the observed reduction. Though still highly toxic in comparison to the non-functional ENp, expression of the EN 9m mutant protein was significantly less toxic than the functional ENp in HeLa cells. Additionally, western blot analysis detected significantly higher steady-state levels of the EN 9m protein in comparison to the functional ENp (Fig. 7), consistent with the previously observed increase in expression of non-functional L1 ORF2 and endonuclease proteins relative to their active counterparts [43, 47]. It is particularly interesting that we did not detect any significant differences in endonuclease activity with the EN or ORF2 proteins containing the S228A mutation when compared to the functional proteins. The S228 residue of the ORF2p endonuclease is predicted to be structurally important [8, 31]. This residue is also highly conserved, and it is even present in its ancient evolutionary ancestor APE1 [8, 31]. Additionally, the S228 residue is in close proximity to the critical H230 residue, the mutation of which is known to eliminate endonuclease activity [8, 30, 31].

Together these results demonstrate that many mutations within the endonuclease domain of ORF2p can be tolerated without substantially impairing endonuclease function and Alu retrotransposition. Although it is plausible that ORF2p mutations may affect L1 and Alu amplification differently, we did not observe any significant variation in the effect of the putative phosphorylation sites mutations tested in both L1 and Alu retrotransposition. The function of the endonuclease domain is predicted to be similar in L1 and Alu retrotransposition, so perhaps a lack of variation is to be expected. The finding that none of the mutations in putative phosphorylation sites that were evaluated in this study eliminated endonuclease activity suggests that, if phosphorylation of these sites occurs, it is not required for endonuclease function in HeLa and 293 cells. However, we cannot rule out the potential impact of these putative phosphorylation sites for ORF2p function in cell types other than the ones tested in this study (HeLa and 293), because cellular kinases often exhibit cell-type-specific expression or activity. All of the putative phosphorylation sites in this study were mutated to alanine, which was a common naturally occurring substitution found in our bioinformatic analysis of the full-length human L1 loci (Additional file 2: Figure S1). Perhaps mutating serine 228 to an alanine was a conservative substitution and did not distort the local protein structure enough to interfere with the function of the neighboring H230. In fact, it is entirely possible that we may have seen different outcomes if any of these sites had been mutated to a different amino acid. It is also worth noting that ORF2p may be phosphorylated at sites other than those investigated in this study.

## Conclusions

Our findings demonstrate that the ORF2 endonuclease domain can tolerate many mutations without significantly impacting its function, either in the context of the full-length ORF2p or in the ENp alone. Despite containing single and multiple point mutations in putative phosphorylation sites, the mutant EN proteins were capable of generating DNA damage and toxicity, and the mutant ORF2 proteins were able to drive Alu retrotransposition with similar efficiency to the functional ORF2p. Even the S228A mutation did not significantly alter endonuclease function, despite its proximity to the catalytic H230 residue and its high conservation among mammalian L1s and related phosphohydrolase ancestors.

## Methods

### Bioinformatic analysis

We searched L1Base to identify mutations in the ORF2 endonuclease domain of full-length human L1 loci [36]. The search criteria were selected to identify L1 loci that contain intact ORF1 and ORF2 sequences (no gaps, premature stops or frameshifts). We found 134 loci fitting these parameters and aligned them by the amino acid sequence of the ORF2 endonuclease domain. All alignments were generated using MegAlign software (DNASTAR v.10.0.1) with human L1.3 as a reference sequence [48]. Alignments using the consensus L1 sequence derived from the 90 intact L1s reported in Brouha *et al.* [1] resulted in the same findings, data not shown. We also aligned the amino acid sequences of L1 ORF2 endonuclease domains from several orders within the Supraprimate clade of mammals. L1 sequences were obtained from RepBase or GenBank for the following species: human [*Homo sapiens*, GenBank: L19088.1, [48]]; chimpanzee [*Pan troglodytes*, GenBank: AY189990.1, [49]]; mouse [*Mus musculus domesticus*, GenBank: AF081104.1, [50]]; rabbit [*Oryctolagus cuniculus*, [51]]; rat [*Rattus norvegicus*, GenBank: U83119.1, [52]]; treeshrew [*Tupaia belangeri*, [51]]; slow loris [*Nycticebus coucang*, GenBank: P08548.1, [53]]; and bonobo [*Pan paniscus*, GenBank: AY189988.1, [49]]. Putative protein phosphorylation sites within the human L1 ORF2 endonuclease domain were identified using NetPhos 2.0 and the ELM prediction tool [38, 39].

### NGS RNA-seq analysis

RNAseq reads were generated using the Illumina platform and total DNase-treated RNA from HeLa cells (TURBO DNase, Ambion). RNA samples were submitted to the University of Wisconsin Genomics Core for selection of polyadenylated RNAs and TruSeq stranded mRNA library preparation. The raw RNAseq data for the HEK293 cell line were obtained from NCBI’s SRA. These files were converted to FASTQ format utilizing fastq-dump:/fastq-dump.2.3.2. The FASTQ files were aligned to the human genome using RSEM, a package that is used for estimating gene and isoform expression levels from data generated through RNA-Seq [54]. A reference genome for the human genome was prepared using the human genome (version GRCh38) and the *rsem-prepare-reference* command. Each FASTQ file was aligned to this generated reference genome using the *rsem-calculate-expression* command. After these samples were aligned to the human genome, a data matrix was generated utilizing RSEM’s EBSEQ using the *rsem-generate-data-matrix* command. A results file was generated from the data matrix using the *rsem-run-ebseq* command. Gene expression from several cellular kinases was analyzed in both cell lines using the genes.results files, generated from the original alignment of each sample to the human genome. The TPM (transcripts per kilobase million) value was obtained by opening genes.results files using Excel and VLOOKUP.

### Plasmids

#### ORF2 putative phosphorylation mutants

Mutations were introduced into a previously reported [26] codon-optimized ORF2 expression plasmid (pBudCE4.1, Invitrogen) using the QuikChange Site-Directed Mutagenesis kit (Stratagene) per the manufacturer’s protocol. Plasmids encoding the full-length ORF2 were generated with the following mutations: S29A; S33A; S37A; S79A; S188A; S228A; S29A/S37A; S79A/T82A; S188A/T189A; S29A/S37A/S228A; S29A/S37A/S188A/T189A; S312A; S335A; and S312A/S335A. The ORF2 11m construct contains the following mutations: S29A/S33A/S37A/S151A/S188A/T189A/T220A/T224A/S228A/S312A/S335A. An ORF2 fragment (amino acids 1–348) containing the aforementioned 11m mutations as well as 5’-HindIII and 3’-AflII restriction sites was synthesized by (GenScript). Site-directed mutagenesis was used to introduce HindIII and AflII restriction sites into the ORF2 expression plasmid. The synthesized fragment containing the 11m mutations was digested with the enzymes listed above and cloned into the similarly digested ORF2 expression plasmid to create the ORF2 11m mutant. Site-directed mutagenesis was used to introduce an inactivating D702A reverse transcriptase [9] mutation into the ORF2 expression plasmid to create the ORF2 RT- construct. Site-directed mutagenesis was used to introduce inactivating D205A endonuclease [8] and D702A reverse transcriptase [9] mutations into the ORF2 expression plasmid to create the ORF2 EN-RT- construct. Primer sequences used for site-directed mutagenesis are shown in Additional file 10: Table S4.

#### EN putative phosphorylation mutants

The codon-optimized endonuclease (EN) and D205A/H230A endonuclease mutant (EN-) expression plasmids (pcDNA3.1/Hygro+, Invitrogen) were previously reported [43]. The ORF2 putative phosphorylation mutant plasmids were used as PCR templates to create the corresponding endonuclease mutant constructs (amino acids 1–239). Plasmids encoding the ORF2 endonuclease domain were generated with the following mutations: S29A; S33A; S37A; S79A; S188A; S228A; S29A/S37A; S79A/T82A; S188A/T189A; S29A/S37A/S228A; S29A/S37A/S188A/T189A or EN 9m (S29A/S33A/S37A/S151A/S188A/T189A/T220A/T224A/S228A). PCR amplification was used to add 5’-NheI and 3’-BamHI restriction sites to the ends of the amplified sequences. The PCR products were subsequently digested with the enzymes listed above and cloned into the similarly digested pcDNA3.1/Hygro+ plasmid.

#### L1 putative phosphorylation mutants

Mutations were introduced into the ORF2 endonuclease domain of a previously reported [55, 56] codon-optimized L1 expression plasmid and a Neo^R^-tagged codon-optimized L1 expression plasmid (pBlueScript II, Stratagene) using site-directed mutagenesis as described above. Plasmids encoding the full-length L1 and the full-length L1 tagged with a Neo^R^ reporter cassette were generated with the following mutations: S29A; S33A; S312A; S335A; and S312A/S335A. Primer sequences used for site-directed mutagenesis are shown in Additional file 10: Table S4. The L1 EN- plasmid, a gift from the Deininger laboratory, contains an inactivating D205A [8] mutation in the ORF2 endonuclease domain of the aforementioned L1 expression plasmid.

The Neo^R^-tagged Alu reporter plasmid used in the retrotransposition assay and the pIRES2-GFP plasmid used in the acute toxicity assay to confer G418 resistance have been previously described [3, 26].

### Cell culture

HeLa and 293-FRT cells were cultured as previously described [43, 45]. Cells were seeded 16–18 h prior to transfection and normal growth media was replaced 3 h post-transfection for all experiments.

### Retrotransposition assays

#### Alu retrotransposition assays

Alu retrotransposition experiments were performed as previously described [3]. For ORF2-driven Alu retrotransposition, 500,000 HeLa cells were seeded per T75 flask and co-transfected the following day with 200 ng of the Neo^R^-tagged Alu reporter plasmid and 200 ng of the ORF2 putative phosphorylation mutant plasmids, using 8 μl of Lipofectamine (Invitrogen) and 4 μl of Plus (Invitrogen). Transfections were performed in duplicate. The experiments shown in Fig. 3 were repeated a minimum of four times and the experiments shown in Fig. 8a were repeated a minimum of three times. For L1-driven Alu retrotransposition, 500,000 HeLa cells were seeded per T75 flask and co-transfected the following day with 200–400 ng of the Neo^R^-tagged Alu reporter plasmid and 200–400 ng of the L1 putative phosphorylation mutant plasmids, using 8 μl of Lipofectamine and 4 μl of Plus. Experiments shown in Fig. 10a were repeated a minimum of two times. For all Alu retrotransposition experiments, selection medium (400 μg/ml G418) was started 24–48 h post-transfection and maintained for 12–14 days to select for G418-resistant colonies representing Alu retrotransposition events. Colonies were fixed and stained with a crystal violet solution (0.2 % crystal violet, 5 % acetic acid, 2.5 % isopropanol). Statistical significance was evaluated using Student’s *t*-test for samples of equal variance; error bars in figures represent standard deviations.

#### L1 retrotransposition assays

L1 retrotransposition experiments were performed as previously described [5]. Approximately 500,000 HeLa cells were seeded per T75 flask and co-transfected the following day with 50–800 ng of the Neo^R^-tagged L1 putative phosphorylation mutant plasmids and 0–350 ng of empty filler plasmid, using 8 μl of Lipofectamine and 4 μl of Plus. Experiments shown in Fig. 10b were repeated a minimum of two times. Selection medium (400 μg/ml G418) was started 24–48 h post-transfection and maintained for 12–14 days to select for G418-resistant colonies representing L1 retrotransposition events. Colonies were fixed and stained with crystal violet solution as above. Statistical significance was evaluated using Student’s *t*-test for samples of equal variance; error bars in figures represent standard deviations.

### Acute toxicity assays

Acute toxicity assay experiments were conducted as previously described, with minor modifications [26, 43]. *ORF2 acute toxicity in HeLa cells:* HeLa cells were seeded at a density of 500,000 cells per T75 flask and transiently co-transfected the following day with 250–400 ng of the ORF2 putative phosphorylation mutant plasmids, 100–250 ng of the pIRES2-GFP plasmid to confer G418 resistance (Neo^R^), and 0–150 ng of empty filler plasmid, using 8 μl of Lipofectamine and 4 μl of Plus. Transfections were performed in duplicate. The experiments shown in Figs. 4a and 8b were repeated a minimum of two times. *ORF2 acute toxicity in 293 cells:* 293 cells were seeded at a density of 125,000 cells per T75 flask and transiently co-transfected the following day with 900 ng of the ORF2 putative phosphorylation mutant plasmids and 100 ng of the pIRES2-GFP plasmid to confer G418 resistance (Neo^R^), using 8 μl of Lipofectamine and 4 μl of Plus. Transfections were performed in duplicate and the experiments shown in Figs. 4b and 8c were repeated twice. *EN acute toxicity:* 500,000 HeLa cells were seeded per T75 flask and transiently co-transfected the following day with 100 ng of the EN putative phosphorylation mutant plasmids, 150 ng of the Neo^R^ expression plasmid, and 150 ng of empty filler plasmid, using 8 μl of Lipofectamine and 4 μl of Plus. Transfections were performed in duplicate, the supplemental experiment shown in Additional file 8: Figure S5 was performed once. For all acute toxicity experiments, selection medium (400 μg/ml G418) was added 24–48 h post-transfection and maintained for 12–14 days to select for G418-resistant colonies. Statistical significance was evaluated using Student’s *t*-test for samples of equal variance; error bars in figures represent standard deviations.

### Chronic toxicity assays

Chronic toxicity assay experiments were conducted as previously described, with minor modifications [27, 43]. The EN putative phosphorylation mutant sequences were cloned into a plasmid which also expresses a gene for hygromycin resistance. Approximately 500,000 HeLa cells were seeded per T75 flask and co-transfected the following day with 200 ng of the EN putative phosphorylation mutant plasmids and 200 ng of empty filler plasmid, using 8 μl of Lipofectamine and 4 μl of Plus. Transfections were performed in duplicate and the experiments shown in Fig. 6 were repeated four times. Hygromycin selection (220 μg/ml) was initiated 48 h post-transfection and maintained for 12–14 days to allow for constant expression of ENp throughout the duration of the assay. Colonies were fixed and stained as described above. Statistical significance was evaluated using Student’s *t*-test for samples of equal variance; error bars in figures represent standard deviations.

### Immunoblot analysis

#### Transfections

To analyze total protein expression, approximately 400,000 HeLa cells or 1.5 million 293 cells were seeded per T25 flask and transfected the following day with 2 μg of the ORF2 or EN expression plasmids, using 8 μl of Lipofectamine and 4 μl of Plus. Cells were harvested approximately 24 h later and western blots were performed as previously described, with minor modifications [43, 57, 58]. The experiments (transfection and subsequent western blot analysis) shown in Fig. 5 were repeated a minimum of four times, the experiments shown in Fig. 7 were repeated three times, and the experiments shown in Fig. 9 were repeated a minimum of three times. The supplemental experiment shown in Additional file 9: Figure S6 was performed once.

#### Total protein harvest

Cells were washed once with 1X phosphate buffered saline (PBS) and lysed in 300 μl TLB-sodium dodecyl sulphate (SDS) buffer [50 mM Tris, 150 mM NaCl, 10 mM ethylenediamine-tetraacetic acid (EDTA), 0.5 % SDS, 0.5 % Triton-X, pH 7.2] supplemented with 10 μl/ml each of the Halt protease inhibitor cocktail (Pierce) and Phosphatase inhibitor cocktails 2 and 3 (Sigma). Harvested cells were sonicated three times for 10 s each at 12 W using a Microson XL-2000 sonicator (Misonix). Cell lysates were collected after centrifugation at 14,000 rpm for 15 min at 4 °C. Total protein concentration was calculated using the Bio-Rad Protein Assay.

#### Western blot

Tris Glycine gels were used for western blot analysis in Figs. 5 and 9. Samples (3.5–15 μg) were boiled for 5 min in denaturing Tris Glycine SDS sample buffer supplemented with β-mercaptoethanol and fractionated on Novex 4 % Tris-Glycine (Invitrogen) gels. Proteins were transferred onto nitrocellulose membranes using the iBlot system (Invitrogen). Membranes were rinsed with PBS-Tween (1x PBS, 0.1 % Tween) and blocked in a mixture containing 5 % non-fat dry milk in 11 ml of PBS-Tween and 4 ml of media collected from NIH-3T3 cells [57]. Membranes were blocked for one hour minimum at room temperature and incubated overnight at 4 °C with custom polyclonal antibodies generated against amino acids 960–973 (NSRWIKDLNVKPKT) of the human L1 ORF2 protein. The ORF2 antibodies were diluted 1:500 in a mixture containing 3 % non-fat dry milk in 11 ml of PBS-Tween and 4 ml of NIH-3T3 media. Membranes were washed and incubated with the secondary HRP-donkey anti-rabbit antibody (Santa Cruz; sc-2317) at a 1:5000 dilution in a mixture containing 3 % non-fat dry milk in 11 ml of PBS-Tween and 4 ml of NIH-3T3 media.

Bis Tris gels were used for western blot analysis in Fig. 7, Additional file 7: Figure S4 and Additional file 9: Figure S6. Samples (3.5–15 μg) were boiled for 5 min in denaturing Laemmli buffer supplemented with β-mercaptoethanol and fractionated on NuPAGE 4–12 % Bis-Tris gels (Invitrogen). Proteins were transferred onto nitrocellulose membranes using the iBlot system (Invitrogen). Membranes were rinsed with PBS-Tween (1x PBS, 0.1 % Tween) and blocked with 5 % non-fat dry milk in PBS-Tween. Membranes were blocked for one hour minimum at room temperature and incubated overnight at 4 °C with polyclonal antibodies generated against the human L1 ORF2 endonuclease domain [42, 43]. Antibodies were diluted in 3 % non-fat dry milk in PBS-Tween as follows: human L1 ORF2 endonuclease domain 1:500; γH2AX (Santa Cruz; sc-101696) 1:100,000; and GAPDH 1:10,000. γH2AX was used as an indicator of DNA damage [34] and GAPDH was used to confirm equal loading of the gel. Membranes were washed and incubated with the secondary antibody, either HRP-donkey anti-goat (Santa Cruz; sc-2020) or HRP-donkey anti-rabbit (Santa Cruz; sc-2317), at a 1:5000 dilution in 3 % milk in PBS-Tween.

All western blots were developed using Clarity Western ECL blotting substrate (Bio-Rad) and the images were captured using a Bio-Rad Gel Doc XR+ imager. The signal intensity of observed bands was quantified before saturation, using Image Lab 4.0.1 software. Statistical significance was evaluated using Student’s *t*-test for samples of equal variance. Error bars in figures represent standard deviations.

## Additional files

Additional file 1: Table S1. Expanded analysis of mutations in the ORF2 endonuclease domain from full-length human L1 loci. Bioinformatic analysis using L1Base [35] revealed numerous mutations in the ORF2 endonuclease domains of 134 intact, full-length L1 loci. The spreadsheet contains our complete query results from L1Base, including the L1 loci locations and the conservation status of several critical amino acid residues within ORF2. (XLSX 211 kb) Additional file 2: Figure S1. Alignment of mutations in the ORF2 endonuclease domain from full-length human L1 loci. Bioinformatic analysis using L1Base [35] revealed numerous mutations in the ORF2 endonuclease domains of 134 intact, full-length L1 loci. The ORF2 endonuclease domain sequences (amino acids 1–239) from these 134 L1 loci were aligned using the Clustal W method. The chromosome and L1Base ID number for each loci is listed in the column on the left. Mutations relative to the L1.3 ORF2 endonuclease domain sequence are indicated by the blue square above the amino acid residues. Dots indicate a match to the L1.3 sequence and mutations are denoted by the single letter amino acid code. (PDF 45 kb) Additional file 3: Table S2. Location of putative phosphorylation sites within the endonuclease domain of L1 ORF2. Putative protein phosphorylation sites within the ORF2 endonuclease domain were identified using the ELM prediction tool [37] and NetPhos 2.0 phosphorylation prediction program [38]. The ELM p-value is a conservative estimate of the probability that an ELM prediction with a given score is a true positive. The significance cut-off is 10e-2. The NetPhos output score is a value in the range of 0.000–1.000 (the higher the score, the higher the confidence of the prediction). (XLSX 10 kb) Additional file 4: Figure S2. Alignment of L1 ORF2 endonuclease domains from several orders within the Supraprimate clade of mammals. The amino acid sequence of the endonuclease domains of species from various mammalian orders were aligned using the Clustal W method. Residues conserved relative to the human L1 endonuclease sequence are shaded in red. Boxes indicate putative phosphorylation sites of interest. (PNG 13086 kb) Additional file 5: Table S3. NGS RNAseq analysis of HeLa and 293 cells. RNAseq reads from HeLa and 293 cells were analyzed to confirm gene expression from several kinases and control genes (ACTB, GAPDH). Expression is reported as TPM, transcripts per kilobase million. (XLSX 10 kb) Additional file 6: Figure S3. Experimental approach for the acute and chronic toxicity assays: A) Acute toxicity assay: Cells are cotransfected with a Neo^R^ expression vector and either the ORF2 or EN construct. Colony formation was assayed after 2 weeks under G418 selection and used as a measure of toxicity. The full-length ORF2 protein contains an endonuclease (EN), z-motif (z), reverse transcriptase (RT) and Cys-domain (Cys). B) Chronic toxicity assay: The Hygro^R^ gene is encoded by the same plasmid as the EN gene. Colony formation was assayed after 2 weeks under hygromycin selection and used as a measure of toxicity. (PNG 295 kb) Additional file 7: Figure S4. Western blot analysis of ORF2 proteins containing mutations in putative phosphorylation sites. Top panel: Representative western blot analysis of total cell lysates harvested from HeLa cells transfected with the indicated ORF2 putative phosphorylation mutant constructs. ORF2 is the functional protein and ORF2 RT- is a non-functional protein containing a mutation in the reverse transcriptase (D702A) domain. Control lanes indicate cells transfected with an empty vector. Lysates were probed with anti-γH2AX antibodies to detect the phosphorylation of histone H2AX in response to DNA damage, top; and anti-GAPDH to serve as a loading control, bottom. Bottom panel: Western blot quantitation. For each sample, the signal detected for γH2AX was normalized to the signal detected for GAPDH. These relative numbers were expressed as a proportion of the relative number detected from the functional ORF2p. (PNG 263 kb) Additional file 8: Figure S5. Acute toxicity assay in HeLa cells transiently transfected with EN putative phosphorylation mutant plasmids. HeLa cells were cotransfected with a Neo^R^ expression vector and the indicated EN putative phosphorylation mutant plasmid. EN is the functional protein and EN- is a non-functional protein containing inactivating mutations (D205A/H230A). Control indicates cells transfected with an empty vector and the Neo^R^ expression vector. Colony formation was assayed after 2 weeks under G418 selection (Y-axis) and used as a measure of toxicity as previously described [26, 42]. (PNG 2274 kb) Additional file 9: Figure S6. Expression of EN putative phosphorylation site mutant proteins in 293 cells generates DNA damage. Representative western blot analysis of total cell lysates harvested from 293 cells transiently transfected with the indicated EN putative phosphorylation mutant plasmids. EN is the functional protein and EN- is a non-functional protein containing inactivating mutations (D205A/H230A). Control lanes indicate cells transfected with an empty vector. Lysates were probed with polyclonal antibodies generated against the human L1 ORF2 endonuclease domain [41, 42]; anti-γH2AX antibodies to detect the phosphorylation of histone H2AX in response to DNA damage; and anti-GAPDH antibodies to serve as a loading control. (PNG 280 kb) Additional file 10: Table S4. Sequences of primers used in this study for site-directed mutagenesis. (XLSX 9 kb)

## Abbreviations

APE1: apurinic/apyrimidic endonuclease 1
DNA DSB: DNA double-strand break
EN: N-terminal endonuclease
HRP: horseradish peroxidase
kDa: kilodalton
L1 or LINE-1: long interspersed element-1
ORFs: open reading frames
PBS: phosphate buffered saline
RT: reverse transcriptase
SVA: SINE-VNTR-Alu elements
