# Supplementary material for: The endonuclease domain of the LINE-1 ORF2 protein can tolerate multiple mutations
Source: Mob DNA. 2016 Apr 19;7:8. doi: 10.1186/s13100-016-0064-x (PMC4837594; doi:10.1186/s13100-016-0064-x)
Supplement: Additional file 2: Figure S1. — Alignment of mutations in the ORF2 endonuclease domain from full-length human L1 loci. Bioinformatic analysis using L1Base [35] revealed numerous mutations in the ORF2 endonuclease domains of 134 intact, full-length L1 loci. The ORF2 endonuclease domain sequences (amino acids 1–239) from these 134 L1 loci were aligned using the Clustal W method. The chromosome and L1Base ID number for each loci is listed in the column on the left. Mutations relative to the L1.3 ORF2 endonuclease domain sequence are indicated by the blue square above the amino acid residues. Dots indicate a match to the L1.3 sequence and mutations are denoted by the single letter amino acid code. (PDF 45 kb) [file 13100_2016_64_MOESM2_ESM.pdf]

- Majority

Sequence	Alignment (positions 1–70)	
Majority	MTGSNSHITILTLNINGLNSAIKRHRLASWIKSQDPSVCCIQETHLTCRDTHRLKIKGWRKIYQANGKQK	
ChrX.seq96	....	70
Chr1.seq97	..............VS...AP.......N...	70
Chr1.seq98	....	70
Chr1.seq99	....M.......................	70
Chr1.seq100	....	70
Chr1.seq101	............................R...	70
Chr1.seq102	....	70
Chr1.seq103	....	70
Chr1.seq104	....	70
Chr1.seq105	............................R...	70
Chr1.seq106	..............V...	70
Chr1.seq107	....	70
Chr1.seq108	..............V....AP.......N.....................I...................	70
Chr1.seq109	....	70
Chr1.seq110	....Y..............................	70
Chr1.seq111	....M.......................	70
Chr2.seq40	..............V.....P.......N....................H....................	70
Chr2.seq41	..............V.....P.......N...	70
Chr2.seq42	....	70
Chr2.seq43	....	70
Chr2.seq44	....	70
Chr2.seq45	..............V.....P.........................M.......................	70
Chr2.seq46	..............V....AP...	70
Chr2.seq47	....	70
Chr2.seq48	....M.......................	70
Chr2.seq49	............................N...	70
Chr3.seq75	...................Y..	70
Chr3.seq76	..............V.....P.......N..........Y..........................R...	70
Chr3.seq77	....	70
Chr3.seq78	....	70
Chr3.seq79	....	70
Chr3.seq82	....	70
Chr3.seq83	....	70
Chr3.seq84	....	70
Chr4.seq23	....	70
Chr4.seq24	...........................E..	70
Chr4.seq25	....S.....M.......................	70
Chr4.seq26	....	70
Chr4.seq27	....	70
Chr4.seq28	....	70
Chr4.seq29	....	70
Chr4.seq30	....	70
Chr4.seq31	....	70
Chr4.seq32	....	70
Chr5.seq55	....	70
Chr5.seq56	....	70
Chr5.seq57	....	70
Chr5.seq58	....	70
Chr5.seq59	....	70
Chr5.seq60	....	70
Chr5.seq61	....	70
Chr5.seq63	....	70
Chr5.seq64	....	70
Chr5.seq65	..............V.....P.......N...........V.............................	70

```
Chr5.seq66    ......................................................................  70
Chr6.seq112   ......N...............................................................  70
Chr6.seq113   ..............V.....P......P..........SF..............................  70
Chr6.seq114   ..............V....AP.................................................  70
Chr6.seq115   ......................................................................  70
Chr6.seq116   .........................K............................................  70
Chr6.seq117   ...................AP.........................M.......................  70
Chr6.seq118   ......................................................................  70
Chr6.seq119   ..............................................M.......................  70
Chr6.seq121   ......................................................................  70
Chr7.seq67    ......................................................................  70
Chr7.seq68    ......................................................................  70
Chr7.seq69    ......................................................................  70
Chr7.seq70    ...........................................I..........................  70
Chr7.seq71    ......................................................................  70
Chr7.seq72    ......................................................................  70
Chr7.seq74    ......................................................................  70
Chr8.seq1     ..............V...............................A.......................  70
Chr8.seq2     ....S.........V....AP.......N.....................I...................  70
Chr8.seq3     ......................................................................  70
Chr8.seq4     ......................................................................  70
Chr8.seq5     ......................................................................  70
Chr8.seq6     ......................................................................  70
Chr9.seq122   ....................................P........F........................  70
Chr9.seq123   ......................................................................  70
Chr9.seq125   ......................................................................  70
Chr10.seq34   ............................N.........................................  70
Chr10.seq35   ........................................................T.............  70
Chr10.seq36   ..............V....AP.................................................  70
Chr10.seq37   ......................................................................  70
Chr10.seq38   ......................................................................  70
Chr10.seq39   ......................................................................  70
Chr11.seq8    ......................................................................  70
Chr11.seq9    ......................................................................  70
Chr11.seq10   ......................................................................  70
Chr11.seq11   ......................................................................  70
Chr11.seq12   ......................................................................  70
Chr11.seq13   ..............................................M.......................  70
Chr11.seq14   ...............H................T..S..................................  70
Chr11.seq15   ...........................................I..........................  70
Chr12.seq50   ...........................................I..K...................R...  70
Chr12.seq51   ..............V....AP.......N.........Y...............................  70
Chr12.seq52   .....A........V....APM......N.................M.......................  70
Chr12.seq53   ..A...Q.........................N...........Q.........................  70
Chr12.seq54   ......................................................................  70
Chr13.seq126  ..............V....AP.......N....................H....................  70
Chr13.seq127  ..............V....AP.......N.........................................  70
Chr13.seq128  ......................................................................  70
Chr14.seq138  ......................................................................  70
Chr14.seq139  ............................N.........................................  70
Chr14.seq140  ....H.........V.....P.......N.................M.......................  70
Chr15.seq133  ......................................................................  70
Chr15.seq134  .........V........H...................................................  70
Chr15.seq135  ......................................................................  70
Chr15.seq136  ....................P.......N.........................................  70
Chr15.seq137  ......................................................................  70
Chr16.seq16   ..............V....AP...Q...N...................................V.....  70
Chr16.seq17   ......................................................................  70
Chr16.seq18   ......................................................................  70
Chr16.seq19   .........T....V....AP......EN.....................................E...  70
```

```
Chr16.seq20    ......................................................................  70
Chr16.seq21    ......................................................................  70
Chr16.seq22    ..........................................V...........................  70
Chr17.seq33    ......................................................................  70
Chr18.seq141   ...................................S..................................  70
Chr18.seq142   ......................................................................  70
Chr18.seq143   ............................N.................M..............S........  70
Chr18.seq144   ..............................................M.......................  70
Chr18.seq145   ....K.........V.....P.................................................  70
Chr20.seq131   .........M..............................V......S......................  70
Chr20.seq132   ..............V.....P.................................................  70
Chr22.seq129   ......................................................................  70
Chr22.seq130   ....................................................S.................  70
ChrX.seq85     ......................................................................  70
ChrX.seq86     ......................................................................  70
ChrX.seq88     ......................................................................  70
ChrX.seq89     .....................................................................Q  70
ChrX.seq90     ......................................................................  70
ChrX.seq91     ..............................................M.......................  70
ChrX.seq92     ......................................................................  70
L1.3 EN1       MTGSNSHITILTLNINGLNSAIKRHRLASWIKSQDPSVCCIQETHLTCRDTHRLKIKGWRKIYQANGKQK  70
```

- Majority

```
Majority       KAGVAILVSDKTDFKPTKIKRDKEGHYIMVKGSIQQEELTILNIYAPNTGAPRFIKQVLSDLQRDLDSHT
                        80        90        100       110       120       130       140
ChrX.seq96     ............................................................H.........  140
Chr1.seq97     ......................................................................  140
Chr1.seq98     ......................................................................  140
Chr1.seq99     ......................................................................  140
Chr1.seq100    ......................................................................  140
Chr1.seq101    ......................................................................  140
Chr1.seq102    ......................................................................  140
Chr1.seq103    ......................................................................  140
Chr1.seq104    ......................................................................  140
Chr1.seq105    ....................................................................Y.  140
Chr1.seq106    ......................................................................  140
Chr1.seq107    .T....................................................................  140
Chr1.seq108    ..............T.......................................................  140
Chr1.seq109    ......................................................................  140
Chr1.seq110    ......................................................................  140
Chr1.seq111    ......................................................................  140
Chr2.seq40     ................................................I.....................  140
Chr2.seq41     ...........................M..........................................  140
Chr2.seq42     ...................................................L..................  140
Chr2.seq43     ......................................................................  140
Chr2.seq44     ......................................................................  140
Chr2.seq45     ...........................M...............M..........................  140
Chr2.seq46     ......................................................................  140
Chr2.seq47     ...................................................L..................  140
Chr2.seq48     ...........................................M..........................  140
Chr2.seq49     ......................................................................  140
Chr3.seq75     ......................................................................  140
Chr3.seq76     .....................................................................I  140
Chr3.seq77     ......................................................................  140
Chr3.seq78     ......................................................................  140
Chr3.seq79     .............L........................................................  140
Chr3.seq82     ..............................................H.......................  140
```

Chr3.seq83	....	140
Chr3.seq84	....	140
Chr4.seq23	....	140
Chr4.seq24	....	140
Chr4.seq25	....	140
Chr4.seq26	....	140
Chr4.seq27	....	140
Chr4.seq28	....	140
Chr4.seq29	....	140
Chr4.seq30	....	140
Chr4.seq31	....H.........	140
Chr4.seq32	....	140
Chr5.seq55	....	140
Chr5.seq56	....	140
Chr5.seq57	....K	140
Chr5.seq58	....	140
Chr5.seq59	....	140
Chr5.seq60	.....................................D................................	140
Chr5.seq61	....	140
Chr5.seq63	....	140
Chr5.seq64	....	140
Chr5.seq65	....T..............	140
Chr5.seq66	....	140
Chr6.seq112	....H..................	140
Chr6.seq113	....	140
Chr6.seq114	....N...	140
Chr6.seq115	....	140
Chr6.seq116	....	140
Chr6.seq117	....	140
Chr6.seq118	....	140
Chr6.seq119	....	140
Chr6.seq121	....	140
Chr7.seq67	....	140
Chr7.seq68	....N..........	140
Chr7.seq69	....	140
Chr7.seq70	....	140
Chr7.seq71	....	140
Chr7.seq72	....	140
Chr7.seq74	....	140
Chr8.seq1	...................................R..................................	140
Chr8.seq2	................S...	140
Chr8.seq3	....	140
Chr8.seq4	....	140
Chr8.seq5	....	140
Chr8.seq6	....	140
Chr9.seq122	....S...	140
Chr9.seq123	....	140
Chr9.seq125	....	140
Chr10.seq34	............................L...	140
Chr10.seq35	....	140
Chr10.seq36	...L..	140
Chr10.seq37	....	140
Chr10.seq38	.............................I.......................................I	140
Chr10.seq39	....	140
Chr11.seq8	....	140
Chr11.seq9	....	140
Chr11.seq10	....	140
Chr11.seq11	....	140
Chr11.seq12	....	140
Chr11.seq13	...........A..	140

```
Chr11.seq14    .........................................................I............  140
Chr11.seq15    ......................................................................  140
Chr12.seq50    ..............Q.......................................................  140
Chr12.seq51    ..............Q.......................................................  140
Chr12.seq52    ..E...........................................................R......A  140
Chr12.seq53    ......................................................................  140
Chr12.seq54    ......................................................................  140
Chr13.seq126   ...........................L..........................................  140
Chr13.seq127   .V....................................................................  140
Chr13.seq128   ......................................................................  140
Chr14.seq138   .........................................P............................  140
Chr14.seq139   ......................................................................  140
Chr14.seq140   ...............Q.........Q............................................  140
Chr15.seq133   ......................................................................  140
Chr15.seq134   ......................................................................  140
Chr15.seq135   ......................................................................  140
Chr15.seq136   .....................................................Y................  140
Chr15.seq137   ......................................................................  140
Chr16.seq16    ......................................................................  140
Chr16.seq17    ......................................................................  140
Chr16.seq18    ......................................................................  140
Chr16.seq19    ............................................F.........................  140
Chr16.seq20    ......................................................................  140
Chr16.seq21    ......................................................................  140
Chr16.seq22    ......................................................................  140
Chr17.seq33    ......................................................................  140
Chr18.seq141   ...................................................L..................  140
Chr18.seq142   ......................................................................  140
Chr18.seq143   ......................................................................  140
Chr18.seq144   ......................................................................  140
Chr18.seq145   .......A.............H................................................  140
Chr20.seq131   ......................................................................  140
Chr20.seq132   ......................................................................  140
Chr22.seq129   ................A.....................................................  140
Chr22.seq130   ......................................................................  140
ChrX.seq85     ......................................................................  140
ChrX.seq86     ..................................................................H...  140
ChrX.seq88     ..........................................H...........................  140
ChrX.seq89     ......................................................................  140
ChrX.seq90     ......................................................................  140
ChrX.seq91     .........................................................D............  140
ChrX.seq92     ......................................................................  140
L1.3 EN1       KAGVAILVSDKTDFKPTKIKRDKEGHYIMVKGSIQQEELTILNIYAPNTGAPRFIKQVLSDLQRDLDSHT  140

- Majority

Majority       LIMGDFNTPLSTLDRSTRQKVNKDTQELNSALHQADLIDIYRTLHPKSTEYTFFSAPHHTYSKIDHIVGS
                        150       160       170       180       190       200       210

ChrX.seq96     ......................................................................  210
Chr1.seq97     ......................................................................  210
Chr1.seq98     ......................................................................  210
Chr1.seq99     ...........I..........................................................  210
Chr1.seq100    ................................................................H.....  210
Chr1.seq101    ......................................................................  210
Chr1.seq102    ......................................................................  210
Chr1.seq103    ......................................................................  210
Chr1.seq104    ......................................................................  210
Chr1.seq105    ......................................................................  210
```

```
Chr1.seq106   ......................................................................   210
Chr1.seq107   ......................................................................   210
Chr1.seq108   ................K.................V...............................R...   210
Chr1.seq109   ...........I..........................................................   210
Chr1.seq110   ......................................................................   210
Chr1.seq111   ...................................................................L..   210
Chr2.seq40    ................R.....................................................   210
Chr2.seq41    ......................................................................   210
Chr2.seq42    ...................................................................L..   210
Chr2.seq43    .....................................................................R   210
Chr2.seq44    .......................N...................................I..........   210
Chr2.seq45    ...........................................................S.......L..   210
Chr2.seq46    ................................Y.....................................   210
Chr2.seq47    ......................................................................   210
Chr2.seq48    ................................................A.....................   210
Chr2.seq49    ............................................N.....................T...   210
Chr3.seq75    .T....................................................................   210
Chr3.seq76    .................T................V...................................   210
Chr3.seq77    ......................................................................   210
Chr3.seq78    ......................................................................   210
Chr3.seq79    ......................................................................   210
Chr3.seq82    ......................................................................   210
Chr3.seq83    .......................................................T..............   210
Chr3.seq84    ......................................................................   210
Chr4.seq23    ......................................................................   210
Chr4.seq24    ................M.....................................................   210
Chr4.seq25    ......................................................................   210
Chr4.seq26    ......................................................................   210
Chr4.seq27    ...................N..................................................   210
Chr4.seq28    ......................................................................   210
Chr4.seq29    ......................................................................   210
Chr4.seq30    ...................................................................L..   210
Chr4.seq31    ......................................................................   210
Chr4.seq32    ......................................................................   210
Chr5.seq55    ................M.....................................................   210
Chr5.seq56    ......................................................................   210
Chr5.seq57    ...................................................I...............L..   210
Chr5.seq58    ......................................................................   210
Chr5.seq59    ......................................................................   210
Chr5.seq60    ...................................................................L..   210
Chr5.seq61    ..........................................A...........................   210
Chr5.seq63    ......................................................................   210
Chr5.seq64    ...........I..........................................................   210
Chr5.seq65    I.................................T................................L..   210
Chr5.seq66    ...E...................N..............................................   210
Chr6.seq112   .......I..............................................................   210
Chr6.seq113   ......................................................................   210
Chr6.seq114   ....................................................L..............L..   210
Chr6.seq115   .............................T................R....................L..   210
Chr6.seq116   ......................................................................   210
Chr6.seq117   ................................Y.V.....................S.............   210
Chr6.seq118   ......................................................................   210
Chr6.seq119   ........................................S.............................   210
Chr6.seq121   ......................................................................   210
Chr7.seq67    ................M.....................................................   210
Chr7.seq68    ......................................................................   210
Chr7.seq69    ......................................................................   210
Chr7.seq70    ......................................................................   210
Chr7.seq71    .................................................K....................   210
Chr7.seq72    ......................................................................   210
```

```
Chr7.seq74     ......................................................................  210
Chr8.seq1      ......................................................................  210
Chr8.seq2      .....................H................................................  210
Chr8.seq3      ......................................................................  210
Chr8.seq4      ......................................................................  210
Chr8.seq5      ......................................................................  210
Chr8.seq6      ......................................................................  210
Chr9.seq122    ......................................................................  210
Chr9.seq123    ......................................................................  210
Chr9.seq125    ......................................................................  210
Chr10.seq34    ..............S...................V...................................  210
Chr10.seq35    ......................................................................  210
Chr10.seq36    I.....................................................................  210
Chr10.seq37    ................M.........................A...........................  210
Chr10.seq38    ..............................D.......................................  210
Chr10.seq39    ................M.....................................................  210
Chr11.seq8     ......................................................................  210
Chr11.seq9     ......................................................................  210
Chr11.seq10    ......................................................................  210
Chr11.seq11    ......................................................................  210
Chr11.seq12    ..................H...................................................  210
Chr11.seq13    F..................................................................L..  210
Chr11.seq14    ......................................................................  210
Chr11.seq15    ......................................................................  210
Chr12.seq50    ..................................................C...................  210
Chr12.seq51    I.................................V................................L..  210
Chr12.seq52    ............................................Q.........................  210
Chr12.seq53    ......................................................................  210
Chr12.seq54    ...................................................................L..  210
Chr13.seq126   F..................................................................L..  210
Chr13.seq127   I..................................................................L..  210
Chr13.seq128   ......................................................................  210
Chr14.seq138   ...................................................................L..  210
Chr14.seq139   ......................................................................  210
Chr14.seq140   ...................................................................L..  210
Chr15.seq133   ......................................................................  210
Chr15.seq134   ......................................................................  210
Chr15.seq135   ...................................................................L..  210
Chr15.seq136   .......................................M........R.....................  210
Chr15.seq137   ...........I..........................................................  210
Chr16.seq16    ................M..................................................L..  210
Chr16.seq17    ......................................................................  210
Chr16.seq18    ......................................................................  210
Chr16.seq19    ................M..................................................L..  210
Chr16.seq20    ......................................................................  210
Chr16.seq21    ...........I..........................................................  210
Chr16.seq22    ......................................................................  210
Chr17.seq33    ...........................................P..........................  210
Chr18.seq141   ......................................................................  210
Chr18.seq142   ................................P.....................................  210
Chr18.seq143   ......................................................................  210
Chr18.seq144   ................................................K.....................  210
Chr18.seq145   ................M..................................................L..  210
Chr20.seq131   ......................................................................  210
Chr20.seq132   ...........I......................T...................................  210
Chr22.seq129   .................................................K....................  210
Chr22.seq130   ......................................................................  210
ChrX.seq85     ......................................................................  210
ChrX.seq86     ......................................................................  210
ChrX.seq88     ......................................................................  210
```

```
ChrX.seq89    ...R..................................................................  210
ChrX.seq90    .............................................H........................  210
ChrX.seq91    ...................................................................L..  210
ChrX.seq92    ................................Y..................A..................  210
L1.3 EN1      LIMGDFNTPLSTLDRSTRQKVNKDTQELNSALHQADLIDIYRTLHPKSTEYTFFSAPHHTYSKIDHIVGS  210
```

- Majority

```
Majority      KALLSKCKRTEIITNYLSDHSAIKLELRI
                       220       230

ChrX.seq96    .............................  239
Chr1.seq97    T............................  239
Chr1.seq98    .............................  239
Chr1.seq99    .............................  239
Chr1.seq100   .............................  239
Chr1.seq101   .............................  239
Chr1.seq102   .............................  239
Chr1.seq103   .............................  239
Chr1.seq104   .............................  239
Chr1.seq105   .............................  239
Chr1.seq106   .............................  239
Chr1.seq107   .............................  239
Chr1.seq108   .............................  239
Chr1.seq109   .............................  239
Chr1.seq110   .............................  239
Chr1.seq111   .............................  239
Chr2.seq40    .............................  239
Chr2.seq41    .T....................M......  239
Chr2.seq42    .............................  239
Chr2.seq43    .............................  239
Chr2.seq44    .............................  239
Chr2.seq45    E............................  239
Chr2.seq46    .............................  239
Chr2.seq47    .............................  239
Chr2.seq48    .............................  239
Chr2.seq49    .............................  239
Chr3.seq75    .............................  239
Chr3.seq76    .................P...........  239
Chr3.seq77    .............................  239
Chr3.seq78    .............................  239
Chr3.seq79    .............................  239
Chr3.seq82    .............................  239
Chr3.seq83    .............................  239
Chr3.seq84    .............................  239
Chr4.seq23    .............................  239
Chr4.seq24    .............................  239
Chr4.seq25    .............................  239
Chr4.seq26    .............................  239
Chr4.seq27    .............................  239
Chr4.seq28    ...............S.............  239
Chr4.seq29    .............................  239
Chr4.seq30    .............................  239
Chr4.seq31    .............................  239
Chr4.seq32    .............................  239
Chr5.seq55    .............................  239
Chr5.seq56    .............................  239
Chr5.seq57    .............................  239
Chr5.seq58    .............................  239
```

Chr5.seq59	. . . .	239
Chr5.seq60	. . . .	239
Chr5.seq61	. . . .	239
Chr5.seq63	. . . .	239
Chr5.seq64	. . . .	239
Chr5.seq65	. . . . V	239
Chr5.seq66	. . . .	239
Chr6.seq112	. . . .	239
Chr6.seq113	. . . . K .	239
Chr6.seq114	. . . .	239
Chr6.seq115	. . . .	239
Chr6.seq116	. . . .	239
Chr6.seq117	. . . .	239
Chr6.seq118	. . . .	239
Chr6.seq119	. . . .	239
Chr6.seq121	. . . .	239
Chr7.seq67	. . . .	239
Chr7.seq68	. . . .	239
Chr7.seq69	. . . .	239
Chr7.seq70	. . . .	239
Chr7.seq71	. . . .	239
Chr7.seq72	. . . .	239
Chr7.seq74	. . . .	239
Chr8.seq1	. . . .	239
Chr8.seq2	. T . . . . T	239
Chr8.seq3	. . . .	239
Chr8.seq4	. . . .	239
Chr8.seq5	. . . .	239
Chr8.seq6	. . . .	239
Chr9.seq122	. . . .	239
Chr9.seq123	. . . .	239
Chr9.seq125	. . . . P	239
Chr10.seq34	. . . .	239
Chr10.seq35	. . . . I . .	239
Chr10.seq36	. . . . I . . . . S . . . F . .	239
Chr10.seq37	. . . .	239
Chr10.seq38	. . . .	239
Chr10.seq39	. . . . T	239
Chr11.seq8	. . . .	239
Chr11.seq9	. . . .	239
Chr11.seq10	. . . .	239
Chr11.seq11	. . . . Q . . .	239
Chr11.seq12	. . . .	239
Chr11.seq13	. . . .	239
Chr11.seq14	. . . .	239
Chr11.seq15	. . . .	239
Chr12.seq50	. . . . C	239
Chr12.seq51	. . . . I	239
Chr12.seq52	. . . .	239
Chr12.seq53	. . . . M	239
Chr12.seq54	. . . . T	239
Chr13.seq126	. . . . A	239
Chr13.seq127	. . . . . . R	239
Chr13.seq128	. . . .	239
Chr14.seq138	. . . .	239
Chr14.seq139	. . . .	239
Chr14.seq140	. . . .	239
Chr15.seq133	. . . .	239
Chr15.seq134	. . . .	239
Chr15.seq135	. . . .	239

Chr15.seq136	....	239
Chr15.seq137	....	239
Chr16.seq16	....	239
Chr16.seq17	....	239
Chr16.seq18	....	239
Chr16.seq19	....................R........	239
Chr16.seq20	....	239
Chr16.seq21	....	239
Chr16.seq22	...........M.................	239
Chr17.seq33	....	239
Chr18.seq141	......................V......	239
Chr18.seq142	....	239
Chr18.seq143	....	239
Chr18.seq144	....	239
Chr18.seq145	.T..........R............K...	239
Chr20.seq131	....	239
Chr20.seq132	....	239
Chr22.seq129	....	239
Chr22.seq130	....	239
ChrX.seq85	....	239
ChrX.seq86	....	239
ChrX.seq88	..........................R..	239
ChrX.seq89	....	239
ChrX.seq90	....N.Y......................	239
ChrX.seq91	....	239
ChrX.seq92	....	239
L1.3 EN1	KALLSKCKRTEIITNYLSDHSAIKLELRI	239

Decoration 'Decoration #1': Hide (as '.') residues that match L1.3 EN1 exactly.
