# Supplementary figures and images for: The endonuclease domain of the LINE-1 ORF2 protein can tolerate multiple mutations
Source: Mob DNA. 2016 Apr 19;7:8. doi: 10.1186/s13100-016-0064-x (PMC4837594; doi:10.1186/s13100-016-0064-x)

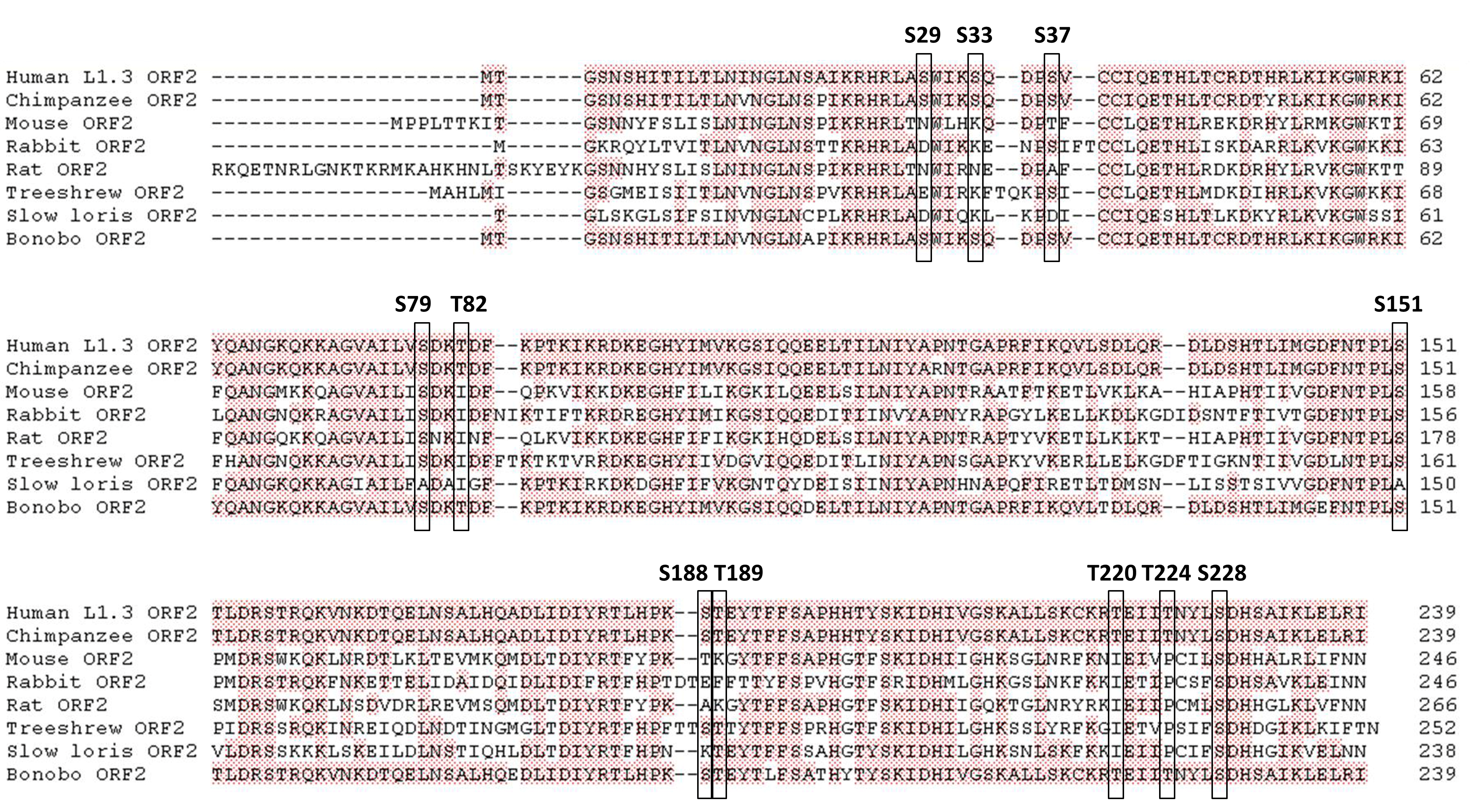

Supplement: Additional file 4: Figure S2. — Alignment of L1 ORF2 endonuclease domains from several orders within the Supraprimate clade of mammals. The amino acid sequence of the endonuclease domains of species from various mammalian orders were aligned using the Clustal W method. Residues conserved relative to the human L1 endonuclease sequence are shaded in red. Boxes indicate putative phosphorylation sites of interest. (PNG 13086 kb) [file 13100_2016_64_MOESM4_ESM.png]

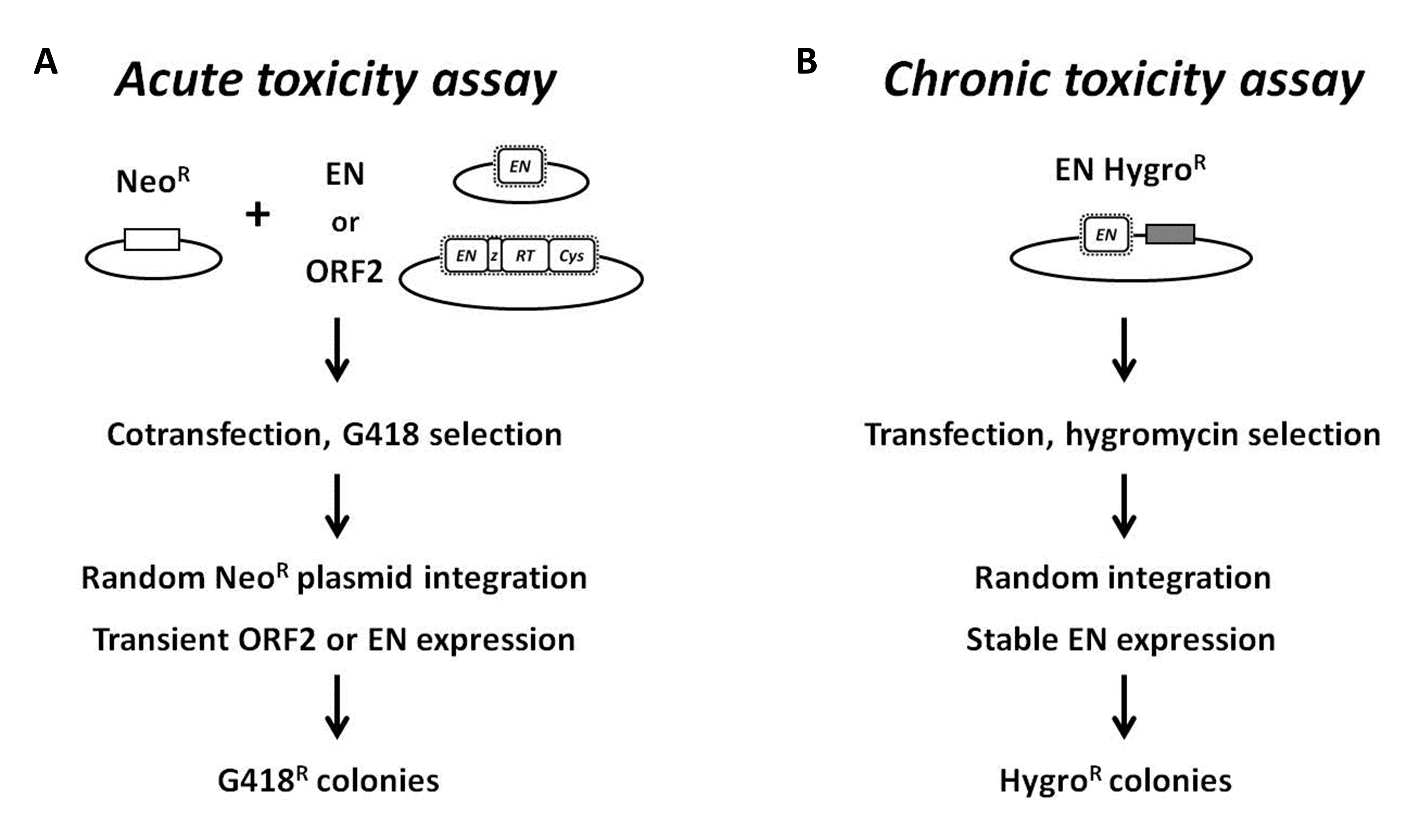

Supplement: Additional file 6: Figure S3. — Experimental approach for the acute and chronic toxicity assays: A) Acute toxicity assay: Cells are cotransfected with a NeoR expression vector and either the ORF2 or EN construct. Colony formation was assayed after 2 weeks under G418 selection and used as a measure of toxicity. The full-length ORF2 protein contains an endonuclease (EN), z-motif (z), reverse transcriptase (RT) and Cys-domain (Cys). B) Chronic toxicity assay: The HygroR gene is encoded by the same plasmid as the EN gene. Colony formation was assayed after 2 weeks under hygromycin selection and used as a measure of toxicity. (PNG 295 kb) [file 13100_2016_64_MOESM6_ESM.png]

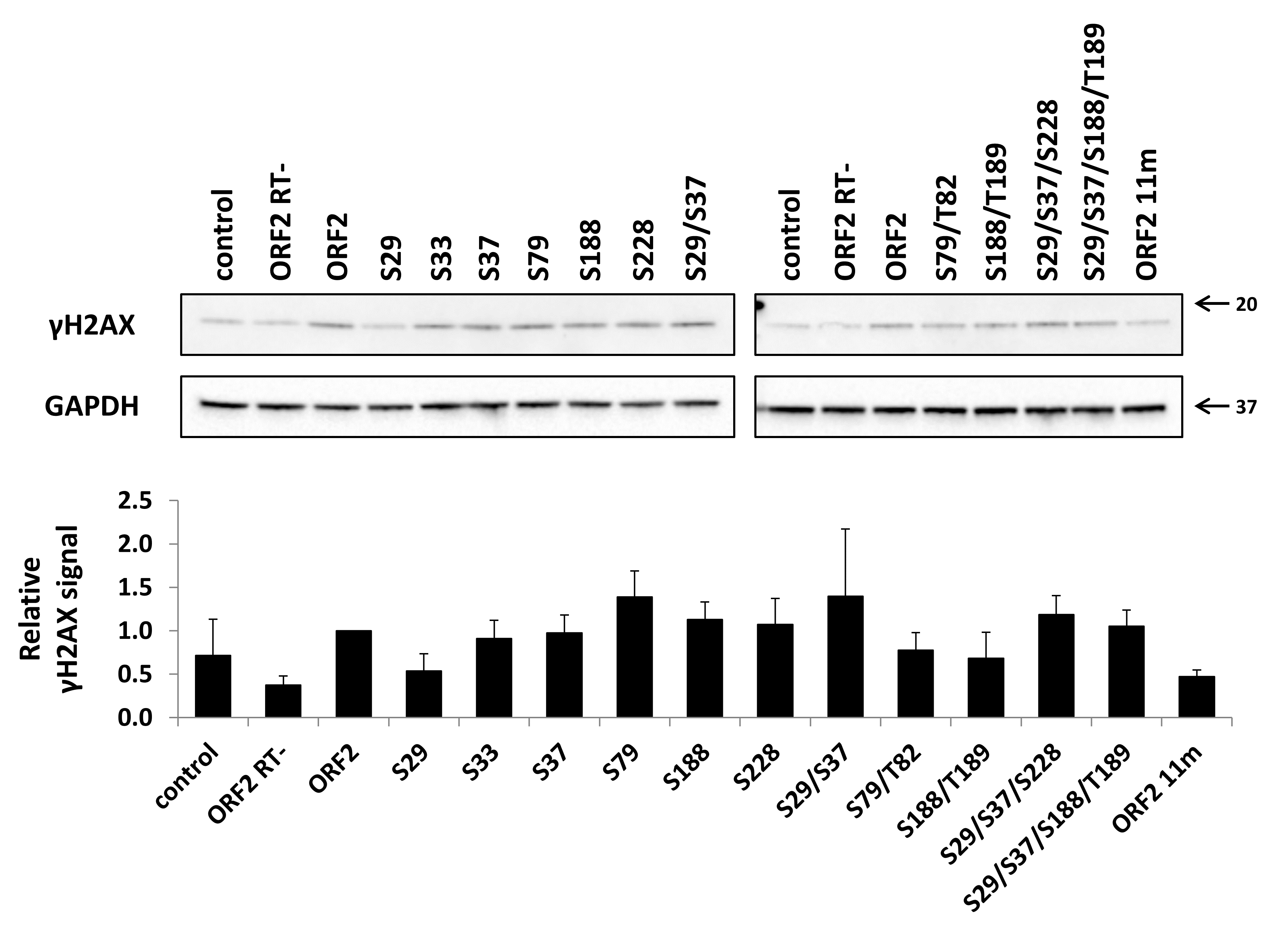

Supplement: Additional file 7: Figure S4. — Western blot analysis of ORF2 proteins containing mutations in putative phosphorylation sites. Top panel: Representative western blot analysis of total cell lysates harvested from HeLa cells transfected with the indicated ORF2 putative phosphorylation mutant constructs. ORF2 is the functional protein and ORF2 RT- is a non-functional protein containing a mutation in the reverse transcriptase (D702A) domain. Control lanes indicate cells transfected with an empty vector. Lysates were probed with anti-γH2AX antibodies to detect the phosphorylation of histone H2AX in response to DNA damage, top; and anti-GAPDH to serve as a loading control, bottom. Bottom panel: Western blot quantitation. For each sample, the signal detected for γH2AX was normalized to the signal detected for GAPDH. These relative numbers were expressed as a proportion of the relative number detected from the functional ORF2p. (PNG 263 kb) [file 13100_2016_64_MOESM7_ESM.png]

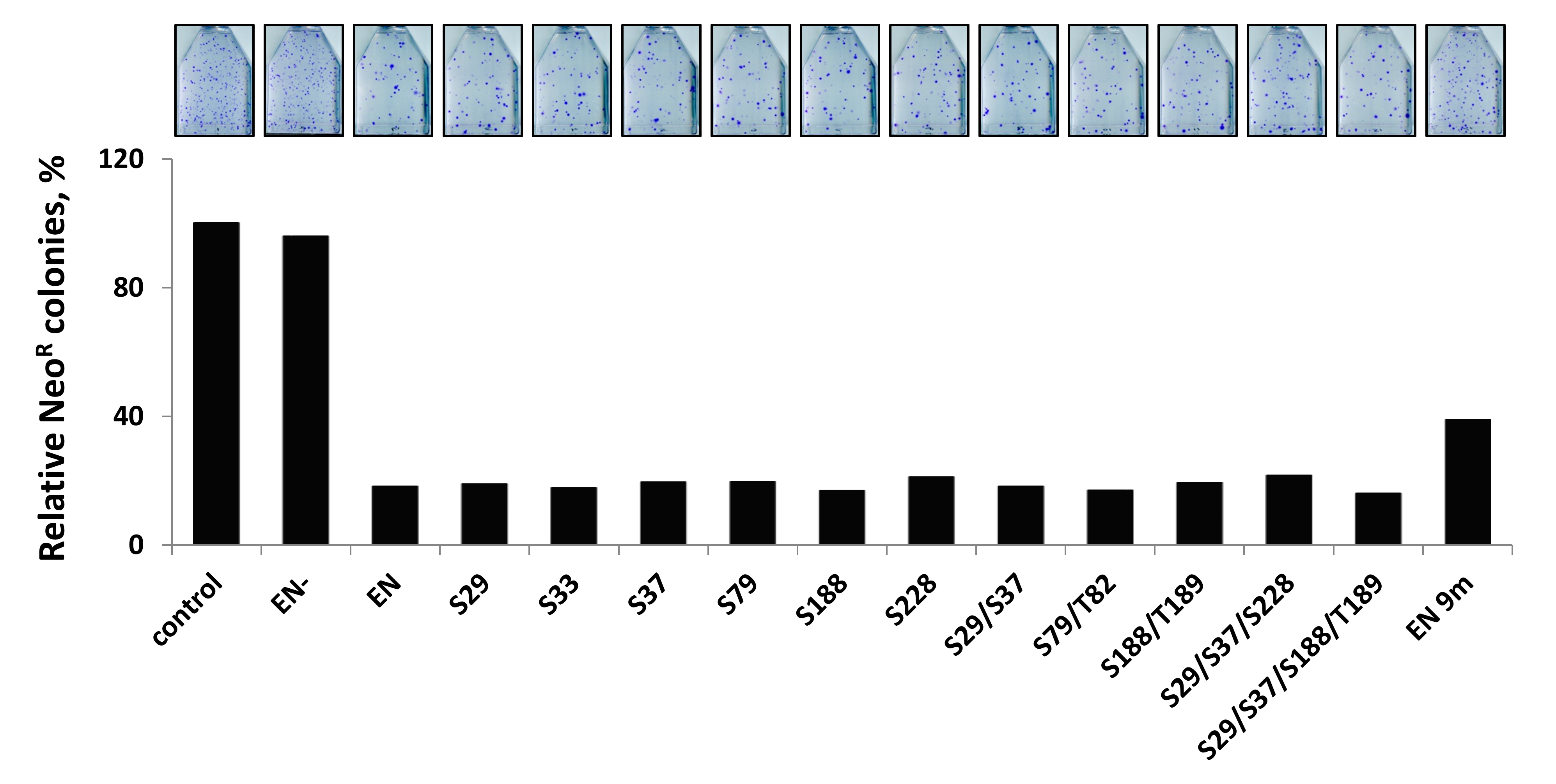

Supplement: Additional file 8: Figure S5. — Acute toxicity assay in HeLa cells transiently transfected with EN putative phosphorylation mutant plasmids. HeLa cells were cotransfected with a NeoR expression vector and the indicated EN putative phosphorylation mutant plasmid. EN is the functional protein and EN- is a non-functional protein containing inactivating mutations (D205A/H230A). Control indicates cells transfected with an empty vector and the NeoR expression vector. Colony formation was assayed after 2 weeks under G418 selection (Y-axis) and used as a measure of toxicity as previously described [26, 42]. (PNG 2274 kb) [file 13100_2016_64_MOESM8_ESM.png]

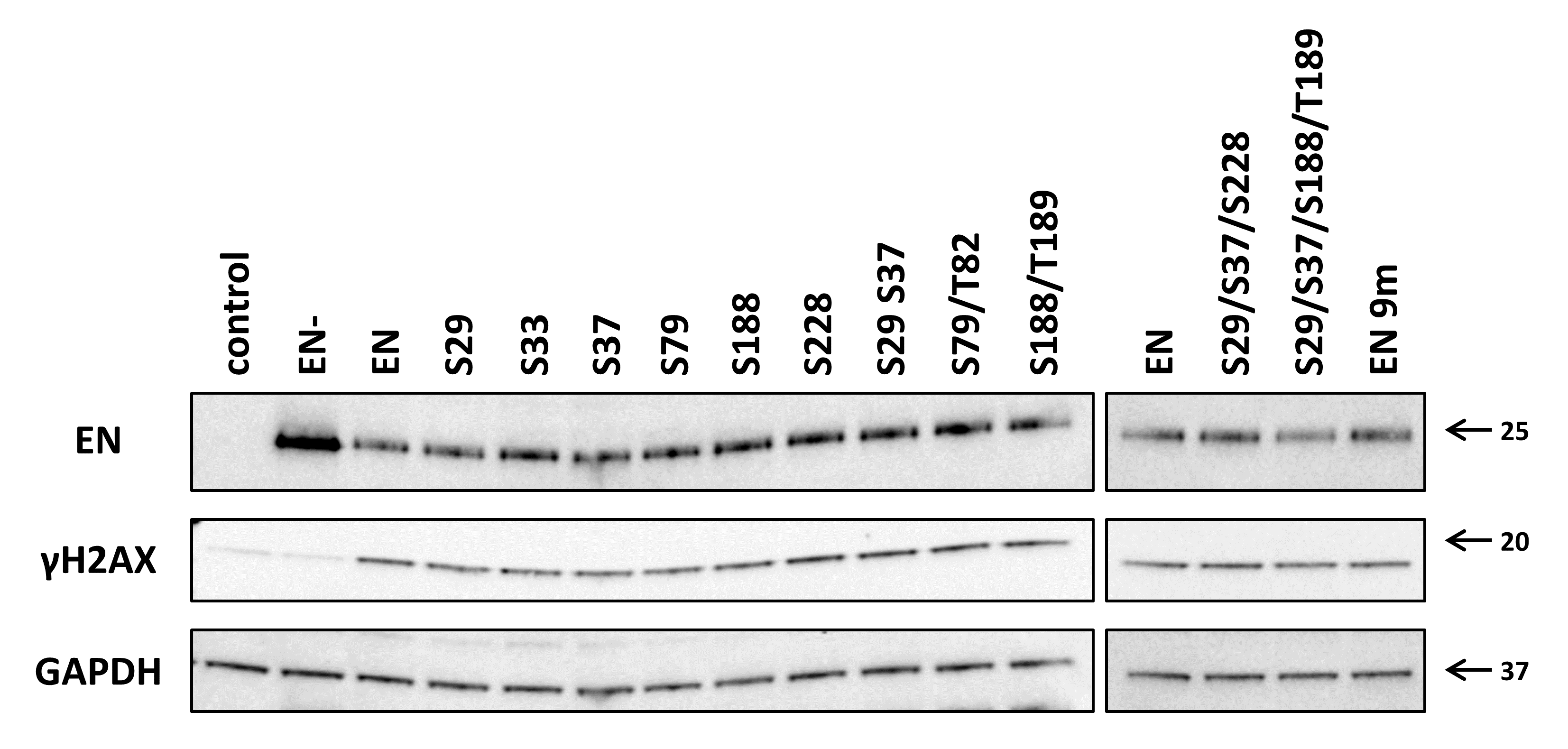

Supplement: Additional file 9: Figure S6. — Expression of EN putative phosphorylation site mutant proteins in 293 cells generates DNA damage. Representative western blot analysis of total cell lysates harvested from 293 cells transiently transfected with the indicated EN putative phosphorylation mutant plasmids. EN is the functional protein and EN- is a non-functional protein containing inactivating mutations (D205A/H230A). Control lanes indicate cells transfected with an empty vector. Lysates were probed with polyclonal antibodies generated against the human L1 ORF2 endonuclease domain [41, 42]; anti-γH2AX antibodies to detect the phosphorylation of histone H2AX in response to DNA damage; and anti-GAPDH antibodies to serve as a loading control. (PNG 280 kb) [file 13100_2016_64_MOESM9_ESM.png]
